# Nanotechnology boosts the efficiency of tumor diagnosis and therapy

**DOI:** 10.3389/fbioe.2023.1249875

**Published:** 2023-07-28

**Authors:** Ying Yang, Mali Lin, Mengfan Sun, Guo-Qiang Zhang, Jianshuang Guo, Jianheng Li

**Affiliations:** Pharmacology and Toxicology Research Laboratory, College of Pharmaceutical Science, Hebei University, Baoding, Hebei, China

**Keywords:** nanotechnology, nanomaterials, tumor imaging, biomarker detection, tumor therapy

## Abstract

The incidence and mortality of cancer are gradually increasing. The highly invasive and metastasis of tumor cells increase the difficulty of diagnosis and treatment, so people pay more and more attention to the diagnosis and treatment of cancer. Conventional treatment methods, including surgery, radiotherapy and chemotherapy, are difficult to eliminate tumor cells completely. And the emergence of nanotechnology has boosted the efficiency of tumor diagnosis and therapy. Herein, the research progress of nanotechnology used for tumor diagnosis and treatment is reviewed, and the emerging detection technology and the application of nanodrugs in clinic are summarized and prospected. The first part refers to the application of different nanomaterials for imaging *in vivo* and detection *in vitro*, which includes magnetic resonance imaging, fluorescence imaging, photoacoustic imaging and biomarker detection. The distinctive physical and chemical advantages of nanomaterials can improve the detection sensitivity and accuracy to achieve tumor detection in early stage. The second part is about the nanodrug used in clinic for tumor treatment. Nanomaterials have been widely used as drug carriers, including the albumin paclitaxel, liposome drugs, mRNA-LNP, protein nanocages, micelles, membrane nanocomplexes, microspheres et al., which could improve the drug accumulate in tumor tissue through enhanced permeability and retention effect to kill tumor cells with high efficiency. But there are still some challenges to revolutionize traditional tumor diagnosis and anti-drug resistance based on nanotechnology.

## 1 Introduction

Cancer is still considered as a major threat to human health (Lu, 2023). According to World Health Organization’s estimates, approximately 10 million individuals pass away globally each year due to cancer. Low income countries and middle-income countries account for a large proportion, and the incidence rate is rising rapidly (Tavallaii et al., 2023).

According to the investigation, malignant tumors can be detected by physical examination, which can prevent their further deterioration in a timely manner. Tumor cells are highly invasive and metastatic, making detection difficult. Most tumor cells are only discovered at a late stage. So far, several imaging techniques have been used for tumor diagnosis, such as magnetic resonance imaging (MRI), computed tomography (CT), and ultrasound imaging (USI). However, these techniques have certain limitations, for example, the low spatial resolution of CT (Sharma et al., 2023). MRI provides high-resolution anatomical images, but its sensitivity is low and tissue penetration is poor (Montiel Schneider and Lassalle, 2017).

Commonly used treatment methods include chemotherapy, surgery, and radiation therapy (Liu et al., 2023). Surgical resection is recognized as the preferred choice for treating solid tumors. Given the limitations of tumor resection, additional methods will be used to assist in targeting tumor residues (Khan et al., 2022). Chemotherapy and radiation therapy are relatively important treatment methods, but there are also many side effects, such as toxicity, drug resistance, and a high probability of late recurrence (Shao et al., 2022; Richardson et al., 2023).

Along with the deep understanding of cancer, more and more studies have begun to focus on the application of nanotechnology for tumor theranostic. The application of nanotechnology in diagnostic and therapeutic applications holds bright prospects. Nanomedicine can enhance the bioavailability of drugs, reduce side effects and extend the duration of drug effectiveness *in vivo* (Teja et al., 2022; Hu et al., 2023). New nanomedicine is constantly being applied in the clinic. Nanotechnology has also made new breakthroughs in cancer diagnosis. Utilizing nanoimaging technology, the sensitivity can be significantly improved, offering the possibility of early detection and timely treatment of cancer. Based on the physical properties of nanomaterials, their applications are becoming more and more extensive. In the diagnosis and treatment of cancer, nanotechnology is seen as a more convenient, highly targeted delivery system with fewer side effects (Gulia et al., 2023). At present, there are still many potential problems with nanotechnology in cancer diagnosis and treatment. This paper reviews the clinical applications of various nanomedicines and their applications in the field of tumor diagnosis and treatment.

## 2 Application of nanotechnology in tumor detection

With the emergence and development of nanotechnology, the small size advantage of nanocarriers can be used to harnessed to enable drugs to engage in nano-level interactions with cells, thereby enhancing the precision of tumor diagnosis (Chen et al., 2023). At present, nanotechnology has made great progress *in vivo* imaging [magnetic resonance imaging (MRI), fluorescence imaging (FI), photoacoustic imaging (PAI), ultrasound imaging (USI)] and *in vitro* detection (exosome).

### 2.1 Nanomaterials imaging *in vivo*

*In vivo* imaging is a powerful strategy in life science research and medical diagnosis. People could better understand the occurrence and development of diseases. The technology is now widely used in the field of oncology. The combination of imaging technology with nanomaterials has led to new breakthroughs in magnetic resonance imaging, fluorescence imaging and photoacoustic imaging.

#### 2.1.1 Magnetic resonance imaging

**FIGURE 1:**
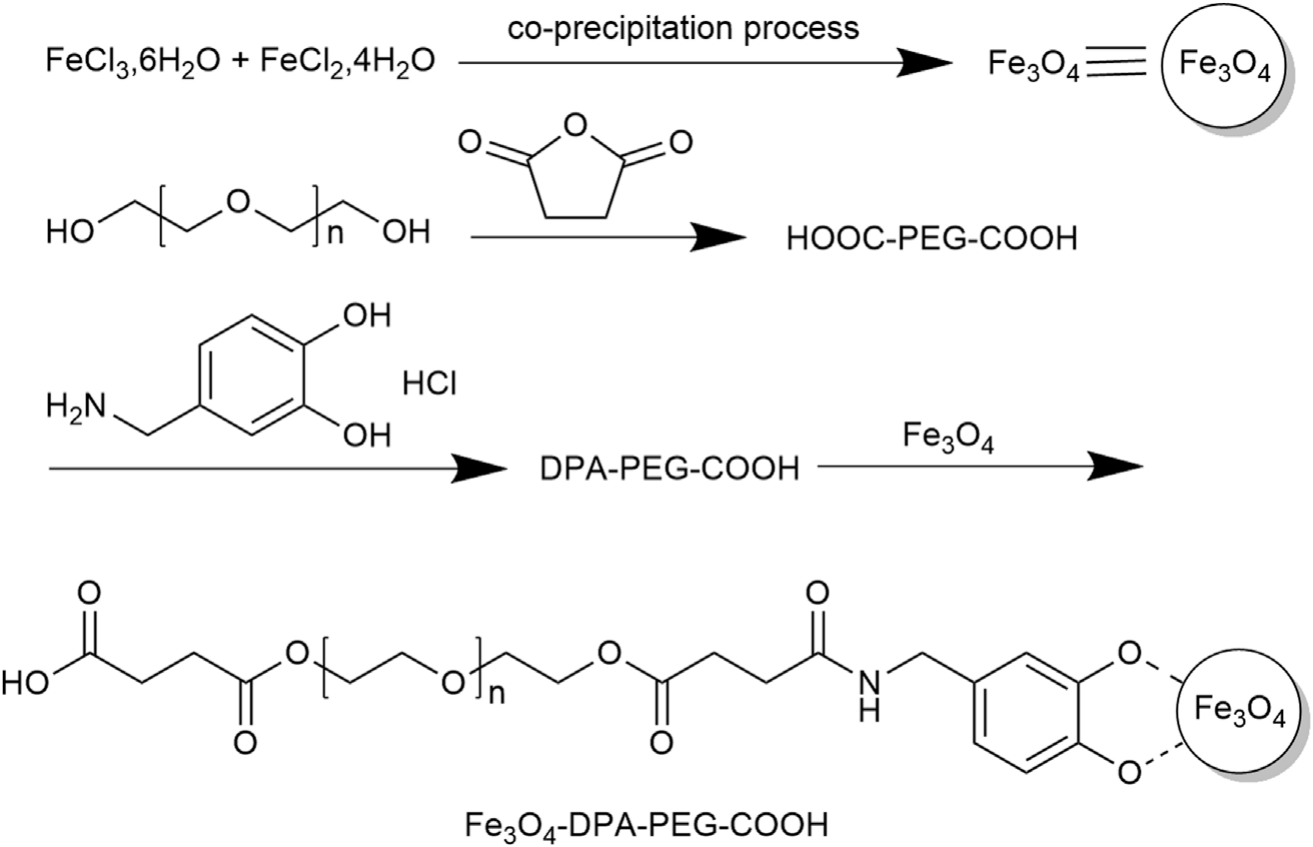
Synthesis of Fe_3_O_4_-DPA-PEG-COOH.

MRI is one of the most commonly used imaging modalities for tumors, but its image resolution is low. The use of contrast agents such as magnetic nanoparticles (MNPs) can effectively solve this problem. MNPs are nanoscale particles centered on a magnetic material and encapsulated in a biopolymer core-shell structure. MNPs has the advantages of both magnetic materials and nanoparticles. They have the advantages of large surface area, strong magnetic responsiveness, high adsorption capacity, good biocompatibility, and easy manipulation by external magnetic fields (Eivazzadeh-Keihan et al., 2021). In the field of tumor-imaging, biomolecule-coated small superparamagnetic iron oxide nanoparticles (SPIOs) and ultramicroscopic paramagnetic iron oxides (USPIOs) have been widely used as magnetic resonance imaging contrast agents for cancer imaging detection. Subsequently, more MNPs for tumor diagnosis have been explored through ongoing in-depth studies. In a study conducted by Rezayan et al. (2016), the impact of nanoparticles used as contrast agents in MRI was explored. SPIOs were synthesized by co-precipitation method according to the synthetic route (Figure 1). The nanoparticles that have undergone modifications exhibit superparamagnetic properties, and they are well-dispersed and highly stable in water. Furthermore, studies have demonstrated that iron oxide nanoparticles modified with amino acids are the most efficient contrast agents for MRI. In a study performed by Eivazzadeh-Keihan et al. (2021), MNPs were subjected to modifications using various materials such as carbon-based nanomaterials, metal oxide nanoparticles, synthetic and natural polymers, antibodies, biomolecules, and amphiphilic polymers. The research highlight that the adsorption mechanism of magnetic nanoparticles is influenced by their size, surface energy and charge. Furthermore, the hydrophilicity of magnetic nanoparticles is a crucial factor in influencing the selectivity of protein binding and separation processes.

#### 2.1.2 Fluorescence imaging

FI is an important modality for tumor detection. It is an indispensable instrument in both physiological and pathological research and drug development. In recent years, nanomaterials have many applications in FI for tumor detection, such as inorganic quantum dots (QDs).

QDs refer to be aggregates composed of a certain number of atoms in a certain way, which can be used to display the distribution and concentration of biomarkers in tissues. QDs have exceptional optical characteristics such as high brightness, strong stability and the ability to perform multiplexing, so they are expected to be a promising optical detector (Cheng et al., 2021). The surface of QDs can be modified to enable object localization imaging. Li et al. (2023d) used a simple strategy to combine Mn: ZnS QDs, and TEMPO-CNFs to detect different types of biological molecules. The biosensor is portable and accurate, make it a valuable tool in fluorescence detection, not only for the targeted substances but also potentially for other compounds in samples. It is expected to provide new ideas for environmental monitoring and biomedical diagnosis.

Carbon quantum dots (CQD) are fluorescent carbon nanoparticles with promising applications in cell imaging and bioimaging due to their photoluminescent properties. Through the use of photobleaching treatment, Huang et al. (2019) synthesized highly stable fluorescent carbon quantum dots (FCQDs) and obtain fluorescence images at different time intervals. The FCQDs exhibited fluorescence 5 min after administration and accumulated at the tumor sites after 3 h. The authors reported that the total accumulation in tumors was observed at 12 h and this extended the imaging period. The researchers also found that the FCQDs efficiently accumulated in tumors, kidneys, and liver based on anatomical images of mouse organs. However, there was no detection of fluorescent signal in organs such as the heart, lung, and spleen. Experiments involving *in vivo* cell imaging and bioimaging demonstrated that the FCQDs exhibited excellent bioimaging properties, low cytotoxicity, and antioxidant activity.

#### 2.1.3 Photoacoustic imaging

**FIGURE 2:**
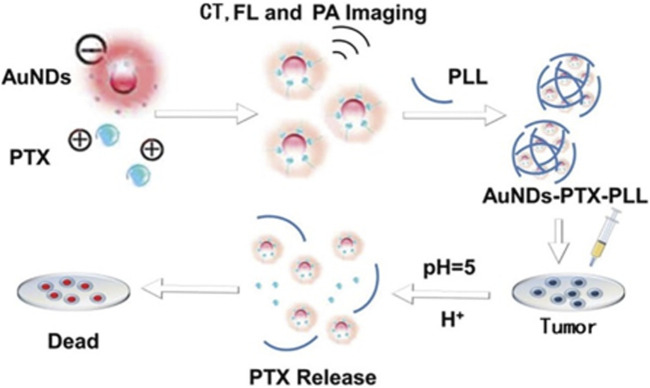
The procedure involved in producing AuNDs-PTX-PLL and its utilization for responsive drug release in chemotherapy applications.

PAI is an advanced technology that integrates ultrasound imaging with optical imaging systems, offering both precise spatial resolution and enhanced contrast. But the resolution of photoacoustic imaging is influenced by many factors. Photoacoustic imaging and gold nanoparticles can be used in combination to achieve more accurate biomedical imaging. Gold (Au) is one of the most chemically stable elements, and Au nanomaterials have good biocompatibility. Gold nanoparticles (AuNPs) are easily adsorbed on other biomaterials (Li H. et al., 2022), and the surface is always covered with protectant molecules. These molecules are used to interact with specific reagents in order to generate certain functional groups with special functions. With this property, AuNPs can be chemically modified and applied to imaging analysis, tumor detection and other fields. In particular, surface passivators, such as protein and peptide capped gold nanoparticles, are widely used for imaging (Shelar et al., 2023). Wang et al. (2022) constructed a core-shell integrated diagnostic and therapeutic nano-system of gold nanodots-paclitaxel-polylysine (AuNDs PTX-PLL) (Figure 2). AuNDs and PTX are encapsulated in PLL, with a size of around 30 nm to facilitate the enrichment of nanoparticles in tumors. Besides, AuNDs can be used for PAI. Given the direct relationship between the concentration of AuNDs and the imaging effect, it becomes feasible to employ them for quantitative analysis. For optimal results, an AuNDs concentration of 1–1.5 mg/mL is recommended, as the signal strength remains relatively consistent within this range. The utilization of AuNDs enables non-invasive and real-time monitoring of drug delivery and release procedures, showcasing their potential for valuable clinical applications.

PAI has become a research hotspot and researchers need to develop ideal nanoparticle transport systems to work with this imaging technology. Indocyanine green (ICG) is a water-soluble tricarbocyanine dye, which is one of the most commonly used PAI agents. Thangavel et al. (2022) added dehydrated ethanol to human serum albumin aqueous solution, and synthesized ICG-PTX nanoparticles by using a desolvation process. ICG-PTX nanoparticles are being used to guide drug delivery targeting CD44-positive non-small cell lung cancer (NSCLC). The size of the nanoparticle is about 315.24 nm and the PDI is less than 0.3. HA (hyaluronic acid) -functionalized-ICG-PTX-loaded nanoparticles had strong PAI average strength signal with nanoparticles excited by light with a wavelength of 806 nm. The maximum PAI intensity was obtained with a detection signal depth of about 10 mm.

#### 2.1.4 Ultrasonic imaging

USI involves the transmission and reception of ultrasound waves, which encounter varying levels of impedance depending on the tissue they pass through, resulting in different degrees of transmission and reflection at the boundaries between different tissues. Examining the reflected signal can provide insights into the internal structure of bodily tissues.

**FIGURE 3:**
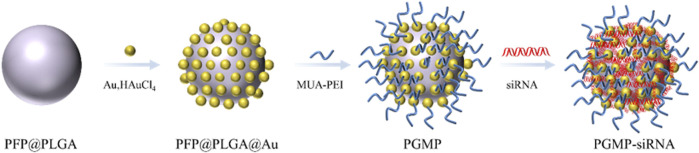
Synthesis of PGMP-siRNA NPs.

**FIGURE 4:**
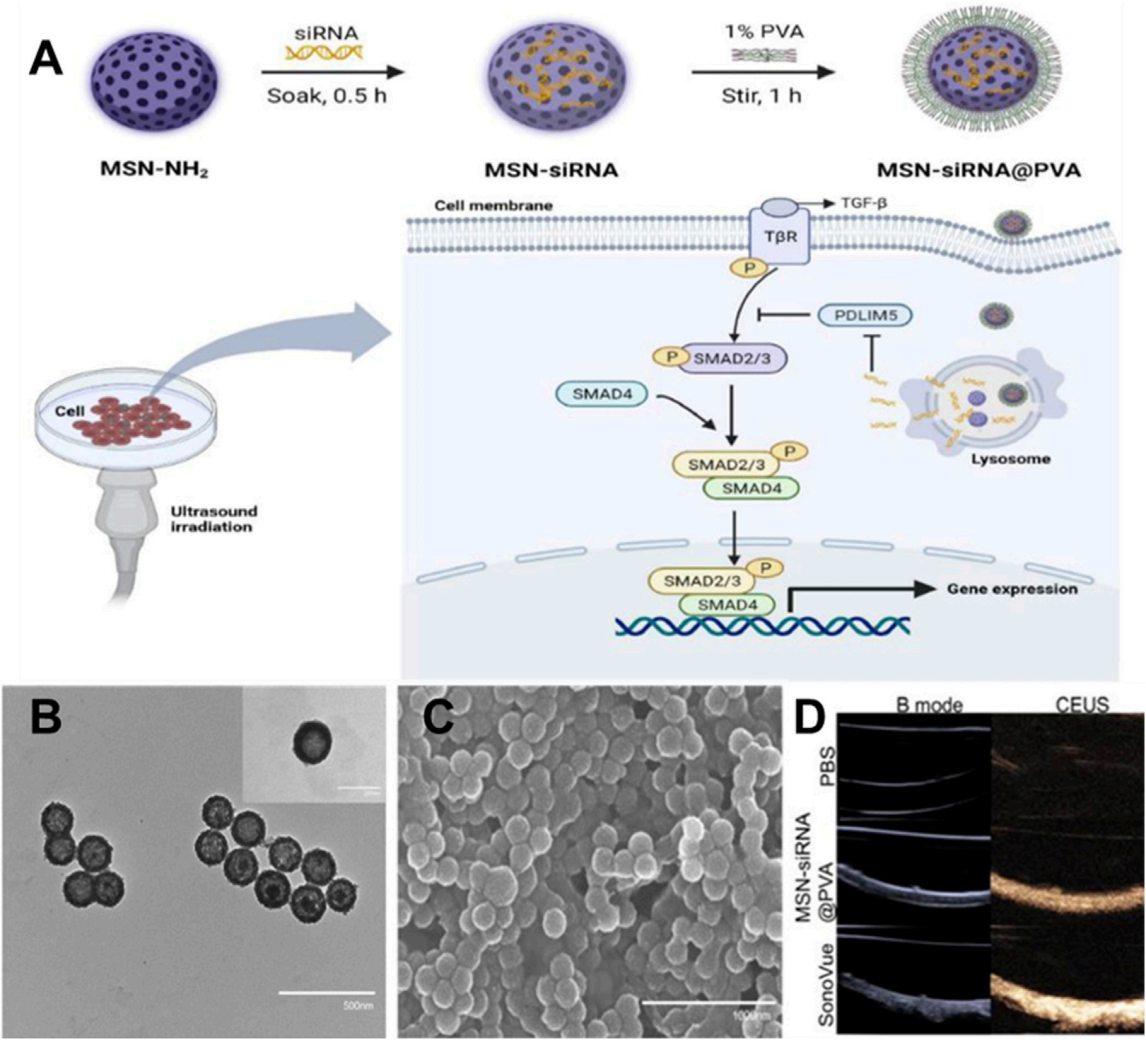
**(A)** Preparation process and antagonistic strategy of siRNA-MSN@PVA NPs. Characterization of MSN-siRNA@PVA nanoparticles. **(B)** TEM **(C)** and SEM images of MSN-siRNA@PVA nanoparticles. **(D)** *In vitro* CEUS imaging of MSN-siRNA@PVA nanoparticles and SonoVue.

Lv et al. (2022) created a type of gold-based nanocarriers called PGMP-siRNA NPs, which were specifically designed to carry small interfering RNA (siRNA) targeting STAT6, with a particle size of about 200 nm (Figure 3). *In vitro* experiments, PGMP-siRNA NPs had strong absorption of light in the wavelength range of 600–900 nm. It can efficiently transform near-infrared light into thermal energy and regulated the temperature by controlling the laser power. In the *in vitro* contrast-enhanced ultrasound (CEUS) mode, the PGMP-siRNA NPs exhibited remarkable improvement in echo intensity. It displayed uniform fine point ultrasonic echo, and the echo was enhanced with the increase of nanocore concentration. Based on their superior ultrasound imaging performance, *in vivo* USI of nanoparticles has been characterized. Tumor sites in PGMP-siRNA NPs-treated mice were significantly enhanced in CEUS mode compared to the control group. These findings provide further validation of the potential of nanoparticles in advancing contrast imaging techniques. Wu et al. (2023) (Figures 4A, B) synthesized a PDLIM5 siRNA delivery system (MSN-siRNA@PVA (polyvinyl alcohol) NPs) based on mesoporous silicon dioxide (MSN) nanobubbles for the treatment of gefitinib resistant NSCLC. The nanoparticles have an approximate size of around 200 nm. In the CEUS test, the MSNsiRNA@PVA NPs echo enhancement is evident, showing a uniform fine-dot high echo. MSN-siRNA@PVA NPs was compared with SonoVue, a commonly used clinical ultrasound contrast agent, to produce similar contrast enhancement at the same concentration (Figure 4C). Therefore, nanoparticles have the potential to be applied in clinical NSCLC diagnosis.

### 2.2 Biomarker detection with nanomaterials *in vitro*

In tumor detection, the conventional approach is to confirm the diagnosis with a biopsy, which is invasive and costly for the patient. Exosomes are ideal analytical targets for liquid biopsies because of the capacity of carrying genetic molecules (Zhang et al., 2021). High quality, low-cost nanosensors are a promising method for exosome protein detection. Compared with other methods, exosomes exhibit excellent performance in biological analysis, including liquid biopsy (Zhang L. et al., 2022; Li et al., 2023b). Nanosensors are typically made of metal or semiconductor nanoparticles whose surfaces are modified with specific biomolecule recognition molecules (e.g., antibodies or nucleic acids) that allow them to bind selectively to the target biomolecule. Kuznetsov et al. (2022) explored a cost-efficient approach to create fluorescent nanosensors utilizing semiconductor nanocrystals. The sensor has excellent photostability and can be used for *in vitro* diagnosis of c-Met positive cells. It exhibits high binding efficacy to the target antigen present on the cell surface and can be utilized for molecular profiling of tumor tissue. The resulting product is expected to be useful for biomarker visualization. Ganesh et al. (2022) designed a self-functionalized nanosensor capable of pathologically identifying metastatic cancer at an early stage and predicting the likelihood of metastatic cancer onset. By attaining a sensitivity rate of 98%, this approach successfully meets the requirements for early detection and prognosis of cancer metastasis through the identification of metastasis-initiating cells (MIC). It demonstrates a remarkable specificity of 99.62% in distinguishing MIC within a diverse population of tumor-initiating cells. Additionally, the predictive accuracy of the MIC assay as a biomarker for metastasis in heterogeneous tumor spheres is 84.6%. In general, this technique is highly relevant for the early identification of metastatic tumors. A near-infrared fluorescent biosensor called α-Fuc-DCM was created by Zhang J. et al. (2022) to enable the quick and ratiometric monitoring of α-L-fucosidase (AFU) activity in cells and mouse models of hepatocellular carcinoma (HCC). The biosensor was found to emit light as a result of the effective catalysis of α L-fucose residues by AFU. The α-Fuc-DCM nanoparticles are a stable biosensor that can be used for the early detection of endogenous AFU activity in a mouse model of HCC. Moreover, the α-Fuc-DCM probe is capable of detecting AFU in serum samples from patients with HCC. This approach offers an effective means of detecting AFU activity, which can be beneficial for the timely diagnosis of HCC.

## 3 Application of nanotechnology in tumor treatment

With the development of nanotechnology, nanomaterials have contributed to the further development of therapeutic tools such as hormone therapy, radiotherapy, transplantation, targeted therapy and immunotherapy. The role of nanomaterials in tumor therapeutic tools can be briefly summarized into two aspects: the first is the direct fabrication of drug molecules into nanoscale particles to address the poor dispersion and high toxicity of existing drugs, such as nanocrystalline drugs; second, nanomaterials are used as drug delivery systems (including albumin paclitaxel, liposome drugs, mRNA-LNP, protein nanocages, micelles, membrane nanocomplexes) for drug delivery.

### 3.1 Drug nanocrystal

In the past few years, an increasing number of scientists have centered their attention on utilizing targeted and controlled-release nanotechnology in the therapy of cancer. with nanocrystalline drugs in the spotlight. Nanocrystals do not require carrier materials. The system is comprised of submicron particles made entirely from drugs, which are arranged in a colloidal dispersion, and there are no limitations on the encapsulation rate. Nanocrystalline drugs often exist as crystalline dispersed particles, and when in liquid form, they are known as nanocrystalline suspensions, which can be defined as nanoparticles made of pure drugs. This is a general formulation method that can improve the delivery efficiency of hydrophobic drugs. This method holds great promise in enhancing the pharmaceutical attributes of active pharmaceutical ingredients that have poor-solubility in water (Ma et al., 2023).

PTX nanocrystals are based on this property to enhance the poor water solubility of paclitaxel. Wei et al. (2015) characterized the saturation solubility, *in vitro* release, stability, and pharmacokinetic properties of PTX nanocrystals. Pharmacokinetic and tissue distribution experiments have shown that PTX nanocrystals can reduce the side effects of cancer therapies and are a promising formulation. PTX nanocrystals have different effects when combined with other drugs. Patel et al. (2014) developed stable PTX nanocrystals with high solubility and dissolution. Clarithromycin was used as a double multi-drug resistance gene cytochrome P450 3A4 inhibitor to enhance PTX penetration and anti-cancer efficacy, and its therapeutic effect on xenografted solid tumor mice was studied. The findings revealed that the nanocrystalline formulation considerably improved the solubility and permeability of PTX, resulting in enhanced therapeutic outcomes. At the same time, co-administration with CLM is a better way to improve the oral bioavailability of taxanes.

Multidrug resistance predisposes to chemotherapy failure, and the fabrication of nanoparticles of celastrol (CST) is an effective solution to overcome the challenges posed by multidrug resistance. The small size and surface modification of nanoparticles of CST can improve the bioavailability and targeting of the drug while reducing drug side effects. Xiao et al. (2022) developed dispersed nanodrugs by directly assembling CST into nanoparticles. The study also investigated the impact of these CST nanoparticles (CNPs) on P-glycoprotein (P-gp) inhibition. Results showed that CNPs were effective in inhibiting P-gp expression and preventing drug efflux by inducing HSF-1 expression and promoting nuclear translocation of HSF-1. The study also found that CNPs promoted apoptosis of newly established doxorubicin (DOX) resistant cell (MCF-7/ADR) by activating the ROS/ERK/JNK signaling pathway, compared to free CST. Moreover, CNPs showed significant inhibitory effects on the proliferation of MCF-7/ADR spheroids, confirming their potential to reverse drug resistance. These findings demonstrated that CNPs have the capability to serve as a translational nanomedicine for addressing drug resistance in cancer treatment. It has also been investigated to synergistically combine ryanodine with doxorubicin Nanocrystals carrier-free for better therapeutic effects through synergistic effects. Through a straightforward and eco-friendly precipitation approach, Xiao et al. (2018) created biocompatible nanoparticles (CST/DOX NPs) that do not require a carrier. The nanoparticles consisted of CST and DOX, two small-molecule drugs that are used clinically, and were developed to enable a synergistic combination chemotherapy strategy that can overcome resistance to DOX. The findings revealed that CST/DOX NPs had a substantial impact on the water solubility of CST and led to an enhanced accumulation of the drug in MCF-7/ADR cells by activating HSF-1 expression and suppressing P-gp expression through the NF-κB pathway. Additionally, the study discovered that CST/DOX NPs triggered apoptosis and autophagy in MCF-7/ADR cells via the ROS/JNK signaling pathway. Furthermore, CST/DOX NPs exhibited significant accumulation and infiltration capabilities in MCF-7/ADR multicellular spheroids (MCs) and had a notable inhibitory effect on MCF-7/ADR MCs. These findings suggest that CST/DOX NPs have great potential as a novel and effective strategy for combating DOX resistance in cancer treatment.

### 3.2 Nanodrug delivery system

Nanoplatforms can be designed as a vehicle to co-deliver multiple drugs, facilitating combination therapy to overcome drug resistance and improve treatment effectiveness (Li et al., 2023c). Nanocarriers can reduce the toxicity level of a drug and improve its therapeutic index. It enables the drug to maintain homeostatic levels for a longer period of time, thus improving efficacy (Chatterjee and Kumar, 2022). Several nanomedicines are gradually entering clinical applications, including nucleic acid-based nanoparticles, polymeric NPs, and classical liposomes (Chatterjee and Kumar, 2022; Zhang et al., 2023). The subsequent parts will discuss the significance of albumin paclitaxel, liposomes, mRNA-LNP, protein nanocages, micelles, Membrane-Nanoparticle Composites, and microspheres in the process of delivering drugs.

#### 3.2.1 Albumin paclitaxel

**FIGURE 5:**
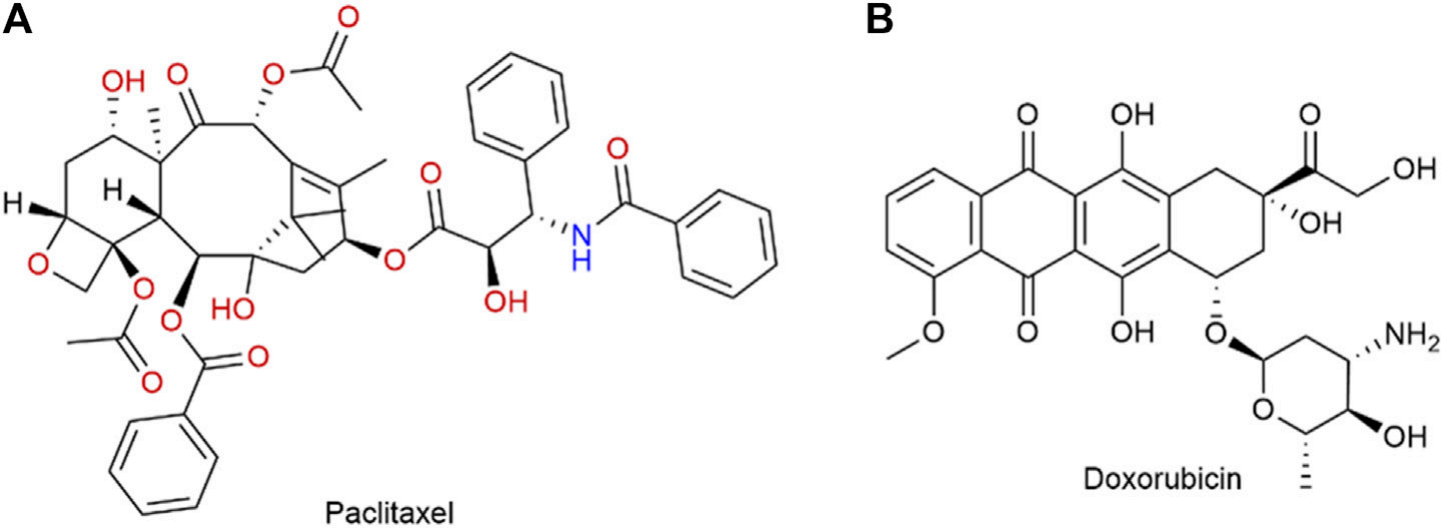
**(A)** Structure of paclitaxel. **(B)** Structure of doxorubicin.

PTX (Figure 5A) is a commonly used chemotherapy drug. In 2005, the FDA approved albumin binding paclitaxel for the treatment of metastatic breast cancer (MBC) (Karthikeyan et al., 2023). It was later approved in 2012 for the treatment of locally advanced or metastatic NSCLC, and in 2013 for metastatic pancreatic cancer (Kundranda and Niu, 2015). Shi et al. (2023) compared the efficacy of solvent-based paclitaxel, liposomal paclitaxel, nanoparticle albumin-bound paclitaxel (Nab-P), and DOX (Figure 5B) in human epidermal growth factor receptor 2 (HER2) positive and HER2 negative breast cancers. It showed that the group containing Nab-P was the most effective in treating breast cancer. Nano-albumin-bound paclitaxel (Abraxane) and solvent-based paclitaxel (i.e., Cre-paclitaxel) have different advantages in clinical applications (Spada et al., 2021). Albumin-bound paclitaxel showed a higher response rate and better tolerance in patients with advanced MBC and NSCLC compared to solvent-based paclitaxel (Yardley, 2013). Yoneshima et al. (2021) compared the therapeutic effects of Nab-P and DOX n patients diagnosed with either squamous or non-squamous NSCLC. Finally, the objective remission rate of albumin-bound paclitaxel was notably higher compared to that of DOX. These findings strongly indicate that albumin-bound paclitaxel demonstrates remarkable efficacy, particularly in patients with advanced NSCLC. The utilization of albumin-bound paclitaxel in the management of advanced NSCLC was also progressing and advancing clinically. And Spigel et al. (2021) evaluated maintenance Nab-P for the treatment of advanced squamous non-epithelial cell lung cancer. A Phase III trial revealed that the combination of Nab-P and carboplatin provided significant benefits for a subgroup of patients with advanced squamous NSCLC. In addition to advanced NSCLC, Nab-P may also have potential benefit for patients with recurrent small cell lung cancer. The progress made in nanotechnology has resulted in the utilization of Nab-P in conjunction with other drugs, with the goal of augmenting its efficacy. A retrospective analysis conducted by Koyama et al. (2018) examined the clinicopathological characteristics of patients who had stage IIIb or IV non-small cell lung cancer (NSCLC) and received a combination treatment consisting of Nab-P and carboplatin (CBDCA). Based on the results of clinical studies, the combination of Nab-P and CBDCA may be considered as a viable treatment alternative for patients with NSCLC and malignant pleural effusion. Multi-drug combination therapies are also emerging. Paglizumab as a targeting antibody modified on the surface of the nanoparticle, which enveloped the carboplatin and albumin paclitaxel by Mazieres et al. (2020). The use of this technique led to a noteworthy enhancement in the overall survival rate, progression-free survival rate, and objective response rate among patients who was untreated metastatic squamous cell carcinoma. The application of paclitaxel combined with Nab-P for clinical cancer treatment is a very promising method, but there are some side effects, such as numbness in the hands and feet, which is one of the more noticeable side effects. The better application of Nab-P to clinical treatment still needs to be improved.

#### 3.2.2 Liposomal drugs

**FIGURE 6:**
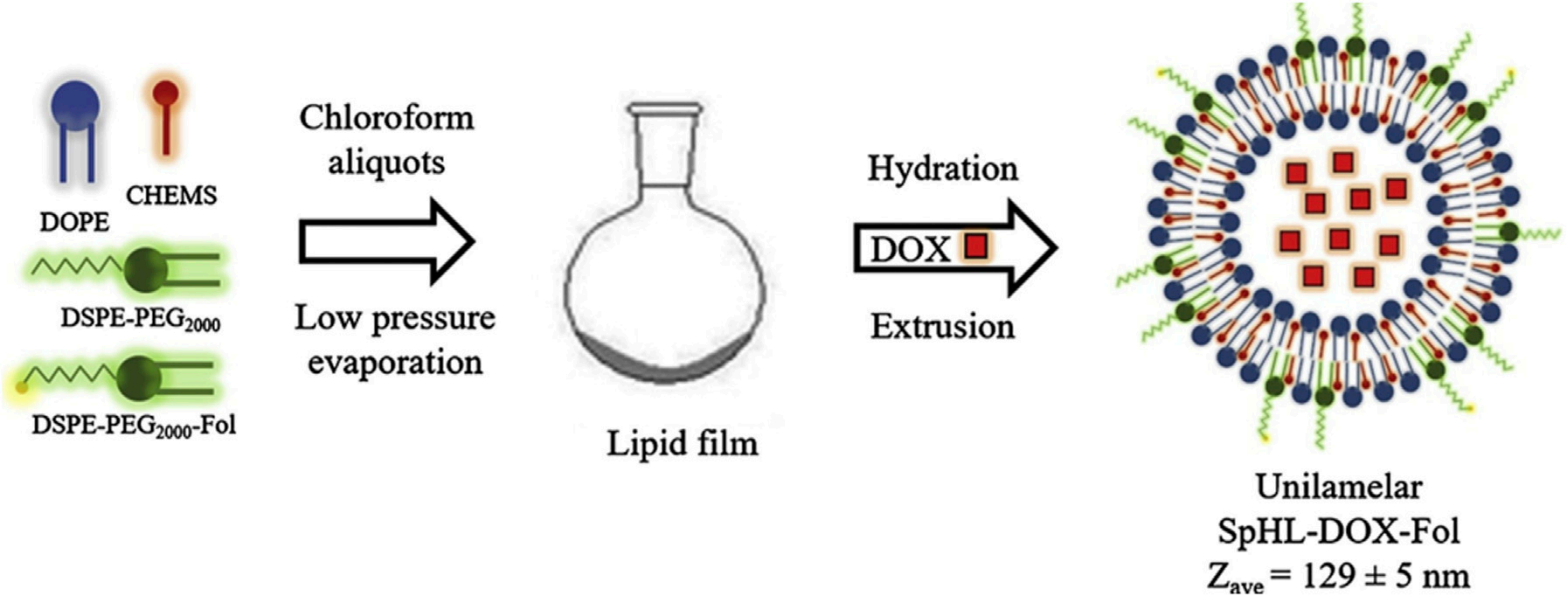
Synthesis of SpHL-DOX-Fol.

Liposomes are phospholipid vesicles composed of single or more concentric lipid bilayer with hydrophobic and hydrophilic cores, are also the most widely used nanocapsules. The therapeutic function of liposomal drugs is strongly related to their lipid composition. In addition, liposomes have been shown to enhance drug dissolution rates and modulate drug delivery (Andresen and Larsen, 2020; Khafoor et al., 2023). Polyethylene glycol doxorubicin liposomes (Doxil) are the first FDA-approved liposomal formulation to form adriamycin sulfate nanocrystals within liposomes during active drug loading (Chakravarty and Dalal, 2018). Researchers are working on the development of novel liposomes, such as long-acting liposomal drugs, cationic liposomes drugs, immune agents, and environmentally sensitive liposomes drugs, in order to enhance drug effectiveness and safety. Modification of empty liposomes with new materials can further improve the targeting ability and safety of liposomes and prolong the half-life of liposomes (He and Tang, 2018). By modifying their surface with hydrophilic polymers, liposomes as drug delivery systems have the advantage of lower leakage rates and longer cycle times. de Oliveira Silva et al. (2019) synthesized long-cycling PH-sensitive folic acid-coated DOX-supported liposomes (SpHL-DOX-Fol). The efficacy of the liposome against tumors was evaluated by conducting both *in vitro* and *in vivo* experiments using a 4T1 breast cancer model (Figure 6). The novel multifunctional nanoplatform demonstrated significant tumor targeting and antitumor effectiveness both *in vitro* and *in vivo*.

Cationic liposomes have a positive surface charge and, unlike their electrically neutral and anionic counterparts. Its low immunogenicity and toxicity, easy quality control and simplicity of preparation make it a common delivery system (Halevas et al., 2019). Various therapeutic payloads can be encapsulated with the help of phospholipid bilayer vesicles of liposome nanoparticles. Doping antibodies (immune liposomes/ILP) on the bilayer surface can amplify the active targeting ability of liposomes. By increasing the affinity for the target cell, the absorption of the drug by the target cell can be selectively enhanced, the off-target effects on normal cells can be mitigated, and the drug efflux can be reduced (Goswami et al., 2022).

The pH-sensitive liposomes are composed of sensitive lipids that remain stable at physiological pH but are unstable under acidic conditions. They possess membrane fusion properties and have attracted attention for achieving rapid drug release in tumor tissue. This function stimulates reactive drug release and decreases drug toxicity. Multi-drug resistance is a key factor in the failure of NSCLC treatment. Wang et al. (2021) prepared porous hybrid paclitaxel-supported liposomes (PPL) containing ambroxol (Ax). It can re-sensitize resistant tumor cells and increase the retention of the drug in the lungs. The combined use of PPL and Ax can achieve outstanding tumor cell killing effect in multiway and multi-aspect, and has a broad range of applications. Liposomal drugs do have clear targeting advantages, but there are also drawbacks, such as the need for sophisticated instruments, high production costs, short half-lives, and the need for newer and better technology.

**FIGURE 7:**
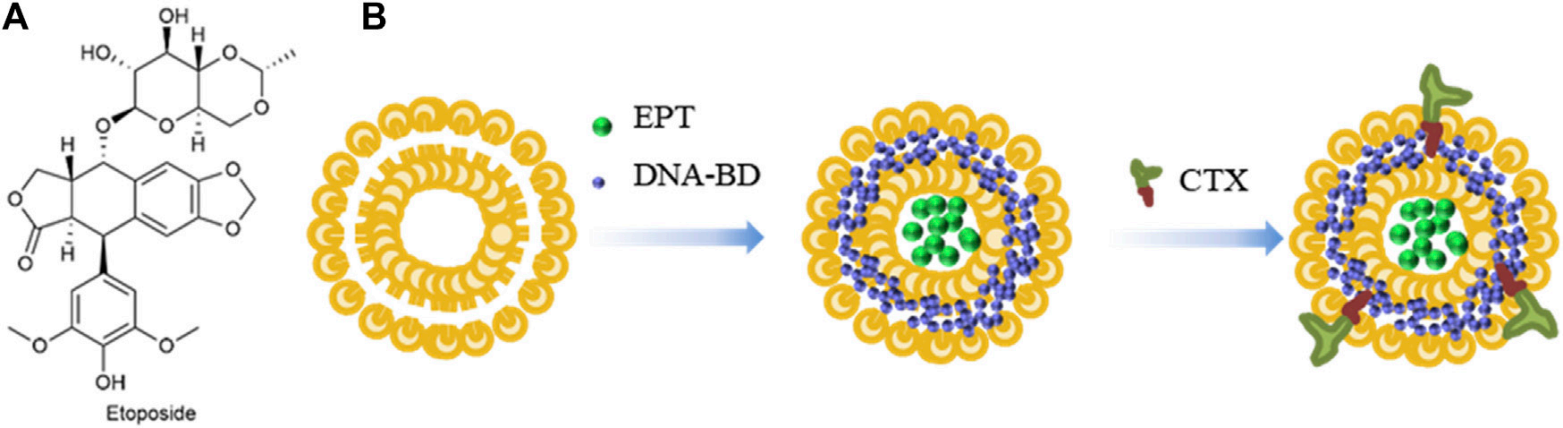
**(A)** Structure of Etoposide. **(B)** Synthesis of liposomes loaded with Etoposide and DNA-BD and coated with Cetuximab.

As shown in Figure 7A, Etoposide (ETP) is a widely used chemotherapy agent and eukaryotic topoisomerase II inhibitor. It breaks down the DNA structure, leading to cytotoxicity (Le et al., 2023). Cetuximab (CTX) is a monoclonal antibody that binds to the epidermal growth factor receptor (EGFR), thereby inhibiting the development of cancer (Baysal et al., 2020). Jha et al. (2020) used liposomes to encapsulate DNA biopoints (DNA-BD) and ETP with CTX as a target agent to modify the surface of nanoparticles. Carboxylated Vitamin-E TPGS (Vit-E TPGS) liposomes loaded with ETP were prepared using an improved solvent injection method, and CTX was coupled to the surface of Vit-E TPGS liposomes by carbodiimide reaction (Figure 7B), with a binding rate of 75%. The size range of liposomes was 140–190 nm, PDI < 0.3, and pH 5.5 was the optimal pH for ETP release. CTX-targeted liposomes have shown strong cytotoxic effects in A549 cells. EGFR-mediated endocytosis enables liposomes to target lung cancer cells and deliver site-specific drugs.

Inhalation administration is a common method for the treatment of lung disease, which reduces the distribution of chemotherapy drugs in the body and alleviates systemic toxicity. In addition, inhalation delivery can improve drug pharmacokinetics and increase drug retention in lungs (Lee et al., 2018). In order to overcome the insufficient pulmonary drug deposition caused by conventional drug delivery methods for treating NSCLC, Sarvepalli et al. (2022) prepared indomethacin liposomes (IND-Lip) by thin film hydration method. The fine particle fraction of nanoparticles was 82.5% ± 0.8%. The median mass values of aerodynamic diameter and geometric standard deviation are 4.3 ± 0.1 µm and 2.3 ± 0.1 µm, in that order. It was shown that the nanoparticles have good atomization behavior and can efficiently enter the tumor and deposit. The cytotoxicity of IND-Lip on three NSCLC cell lines (H460, H1299, and A549) was detected by MTT assay, and the results showed that the IC_50_ value of IND-Lip was 1.5–4 times lower than that of ordinary IND. The efficacy of IND-Lip was better than that of ordinary IND. *In vitro* 3D globular cell administration experiments, the IND-Lip at 200 µM could reduce the original tumor volume by approximately 20%.

**FIGURE 8:**
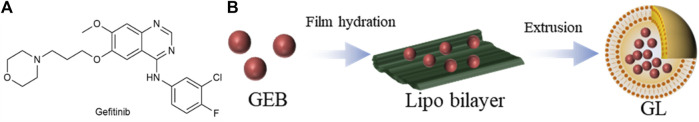
**(A)** Structure of gefitinib. **(B)** Synthesis of GL.

Gefitinib (GEB) (Figure 8A) is a selective EGFR-tyrosine kinase inhibitor (EGFR-TKI) (Lai et al., 2019). In light of the severe side effects of GEB and the limited effectiveness of using single drugs, Hu et al. (2020) opted to utilize a thin film dispersion method that incorporated anhydrous ethanol as a lipid solvent to produce liposomes containing GEB, denoted as GL (Figure 8B). GL has a particle size of about 190 nm, the PDI is 0.248, and the IC_50_ value is 29.63 μg/mL. When the ratio of phospholipid to cholesterol was 3, the highest encapsulation rate of 60.26% was obtained. The drug release time of GL is about 12 h, and the cumulative release rate reaches 80%, which indicates that the GL nanocomposite structure has a certain stability and can effectively prolong the drug action time. In *in vivo* anti-cancer model experiments, the GL group consistently maintained a tumor size of approximately 100 mm^3^, which was significantly smaller than that of the PBS group. The mean tumor weight was 0.066 g in the GL group and 1.167 g in the PBS group. Nanoparticle liposomal carriers have promising applications in the treatment of lung cancer.

Bevacizumab (Beb) is an antibody targeting vascular endothelial growth factor and it is the first-line drug for metastatic colorectal cancer. As the first drug approved to target tumor angiogenesis, Beb is also being used to treat other cancers, including NSCLC and renal cell carcinoma (Muhsin et al., 2004). Zhang J. et al. (2022) prepared cationic liposomes containing anticancer drugs Geb and Beb by thin-film hydration method. The diameter of the nanoparticle is approximately 160 nm. Dialysis was utilized to evaluate the release properties of Geb and Beb. According to the *in vitro* release kinetics curves obtained in a PBS buffer at pH 5.0 and pH 7.4, it can be observed that the nanoparticles exhibit a pH-responsive behavior for the release of Geb and Beb. In addition, the drug release rate of MnO_2_-PDA@Lipo@Geb@Beb nanomaterial is faster in high glutathione (GSH) conditions than in non-redox conditions, indicating that the nanoparticles have GSH response properties. MnO_2_-PDA@Lipo@Geb@Beb also significantly inhibits tumor blood vessel formation. The results obtained during experimentation in a mouse tumor model validated that it displayed a more pronounced impact in inhibiting the growth of NSCLC cells compared to the unbound pharmaceutical agent.

#### 3.2.3 mRNA-LNP

Messenger RNA (mRNA) is an emerging and intriguing form of tumor vaccine that provides antigen delivery and intrinsic immune activation-mediated co-stimulation in a spatiotemporally synchronized manner. mRNA vaccines are designed to instruct cells to express almost all essential proteins in host cells and tissues. These proteins may have a preventive effect, which play the therapeutic role in treating multiple diseases (Granados-Riveron and Aquino-Jarquin, 2021). Lipid nanoparticles (LNP) are simple, biocompatible, inexpensive, and versatile nanocarriers, which can be used to design multifunctional nanosystems (Granja et al., 2023). Initially, researchers used LNP as a vector to encapsulate full-length mRNA encoding novel coronavirus stinger proteins. Immune cells are able to recognize and ingest viral mRNA. Protein antigens can be efficiently expressed by directed mRNA. Tahtinen et al. (2022) discovered that the RNA and lipid formulation of RNA vaccines played a crucial role in the induction of interleukin 1 (IL-1) cytokine production in human immune cells. The IL-1 pathway is known to be vital for initiating innate signaling in response to RNA vaccines. Interestingly, specific lipids used in vaccine formulations containing N1-methylpseudouracil modified RNA unexpectedly increased this effect, thereby enhancing the activation of toll-like receptor signaling. These findings highlight the significance of the lipid component of LNP in stimulating immune responses to mRNA vaccines.

To achieve effective cancer vaccines, it is essential to generate a durable and high-quality CD8^+^ T cell response. Although systematic vaccination is widely recognized as the most effective strategy, it is crucial to meticulously adjust the composition of the LNP carrier to ensure the successful delivery of mRNA to antigen-presenting cells (Bevers et al., 2022). The delivery of mRNA-encapsulated LNPs to the lungs is not without its challenges. One of the difficulties is that the nebulization process of LNPs can subject them to shear stresses caused by air dispersion, air jets, ultrasound and vibrational nets. This shear stress can lead to the clustering or leakage of LNPs, which can disrupt cross-cellular transport and endosomal escape. To overcome this problem, Miao et al. (2023) optimized the LNP formulation, nebulization method, and buffer system to keep the stability of LNPs during nebulization and preserve mRNA efficiency.

As a potential substitute for traditional drugs, mRNA-based therapies show great promise in cancer treatment. By encoding inactivated genes, mRNA therapy can restore the function of tumor suppressor genes that have been deleted or inactivated, thus inhibiting the growth of tumors that result from such mutations (Zong et al., 2023). Additionally, mRNA is translated in the cytoplasm, allowing temporal control of immunotherapy. The benefits of mRNA immunotherapies have made them an attractive option for clinical applications, including cancer treatment modalities such as chimeric antigen receptor T cell (CAR-T) therapy and tumor infiltrating T cell therapy. The use of LNPs as a delivery platform is particularly promising, as they are able to efficiently deliver mRNA to T cells while minimizing toxicity during *in vivo* transfection (Patel et al., 2022). LNPs can be engineered to respond to external stimuli, such as the presence of tumors or the acidic environment of the endosome, thereby releasing mRNA in a targeted manner. Furthermore, LNPs can undergo modifications with targeting ligands and other substances to augment their affinity for overexpressed target proteins, like HER2 on cancer cells, and facilitate enhanced absorption by the intended tissue (Wang et al., 2023). The effectiveness of mRNA delivery through LNPs is heavily influenced by the lipid composition of the LNP, with the ionizable lipids being the primary determinant of delivery efficiency (Zeng et al., 2023). Snow et al. (2022) developed a new minor histocompatibility antigen (mHA) LNP vaccine using UNC-GRK 4-V as a target. They hypothesized that the vaccine might induce an antigen-specific immune response. The result showed that the UNC-GRK 4-V mRNA LNP vaccine based on ionizable amino lipids (SM-102) could trigger a robust antigen-specific T cell response. This could be an effective treatment to reduce the recurrence rate of tumors after allografts. Zeng et al. (2023) developed a LNP to deliver mRNA to bone marrow dendritic cells and evaluated its ability to activate T cells. The researchers confirmed that the LNP proved to be efficient and well-tolerated in delivering mRNA, demonstrating its exceptional suitability as a carrier for mRNA vaccines. Four different LNP formulations were identified, which effectively induced T cell expansion and cytokine production, and these formulations will be subjected to additional *in vivo* investigations as part of the efforts to create cancer vaccines.

#### 3.2.4 Protein nanocages

Protein nanocages have attracted significant attention in different areas of nanomedicine owing to their inherent characteristics, such as biocompatibility, biodegradability, high structural stability, and easily modifiable surfaces and internal cavities (Wang and Douglas, 2021). Protein-based nanocages have great potential as diagnostic systems in cancer treatment. Protein-based nanocages, with their physiological properties and bioengineering versatility, make them a promising candidate for clinical applications. The integration of imaging and therapeutic functions within ferritin nanocages is currently considered the most promising and attractive frontier in cancer diagnostic and therapeutic approaches (Truffi et al., 2016). Nanocages based on ferritin contain anthracyclines, including adriamycin, which is widely used in cancer treatment to inhibit cell proliferation by blocking isomerase 2 (Palombarini et al., 2020). Protein cage nanoparticles such as ferritin, coated insulin, and virus-like particles (VLPs) have significant applications in vaccine development and imaging. These protein-based nanoparticles offer unique advantages compared to chemically synthesized nanomaterials as drug or vaccine carriers. They have many applications in vaccine development, for example, where they can be efficiently targeted and retained in the lymph nodes. Protein nanoparticles can enhance the immune response in various ways, such as facilitating efficient uptake by antigen-presenting cells and forming multiple connections with B cell receptors, thereby improving immunogenicity. Li et al. (2021) developed a new peptide antigen using VLPs from the phage P22 vaccine delivery system. The peptide antigens were expressed by fusion expression on the protein fraction of P22 VLPs. The produced vaccine particles stimulated a significant production of specific antibodies, with titers reaching 5.0 × 10^5^. These findings suggest that P22 VLPs are vehicles for delivering peptide antigens in vaccines and present a versatile and straightforward technology platform for developing personalized therapeutic oncology vaccines.

#### 3.2.5 Micelles

Micelles are mostly spherical structures formed by association of amphoteric molecules at specific temperatures and appropriate concentrations, with particle sizes ranging from 5–100 nm. The micelle’s center is created by hydrophobic segments of amphoteric molecules, while the micelle’s outer layer is made up of hydrophilic fragments (Milovanovic et al., 2017). Many drugs made from micelles have been applied in clinical (Sun et al., 2018), and micelles are mostly used to deliver insoluble chemotherapy drugs in cancer treatment (Ghosh and Biswas, 2021).

**FIGURE 9:**
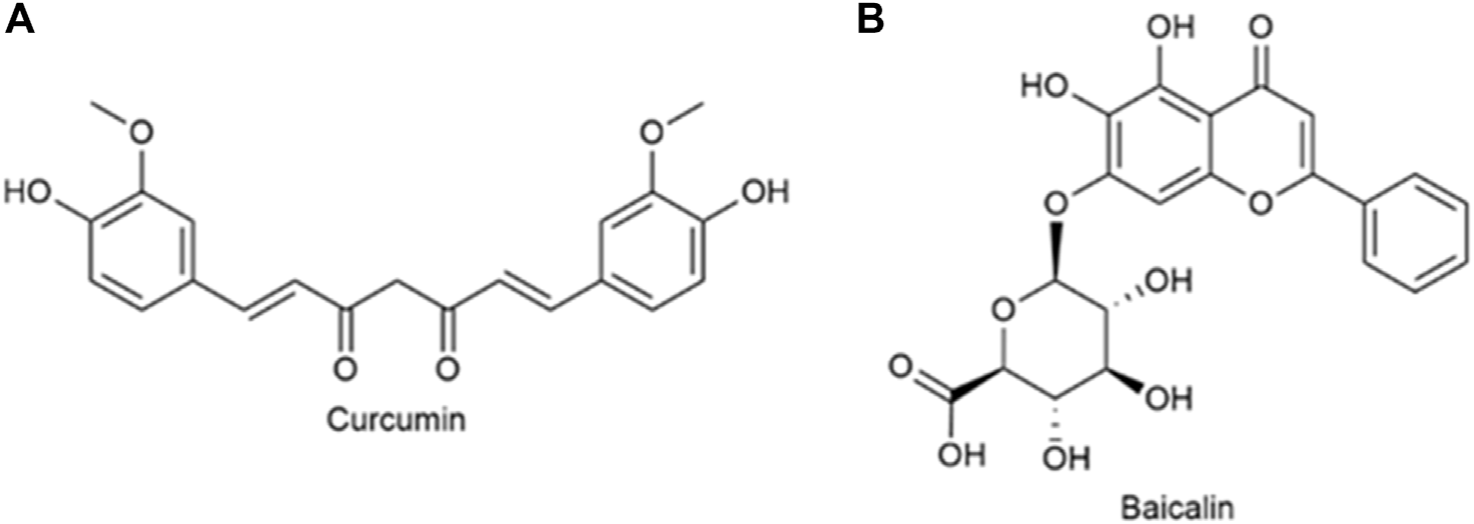
**(A)** Structure of curcumin. **(B)** Structure of Baicalin.

**FIGURE 10:**
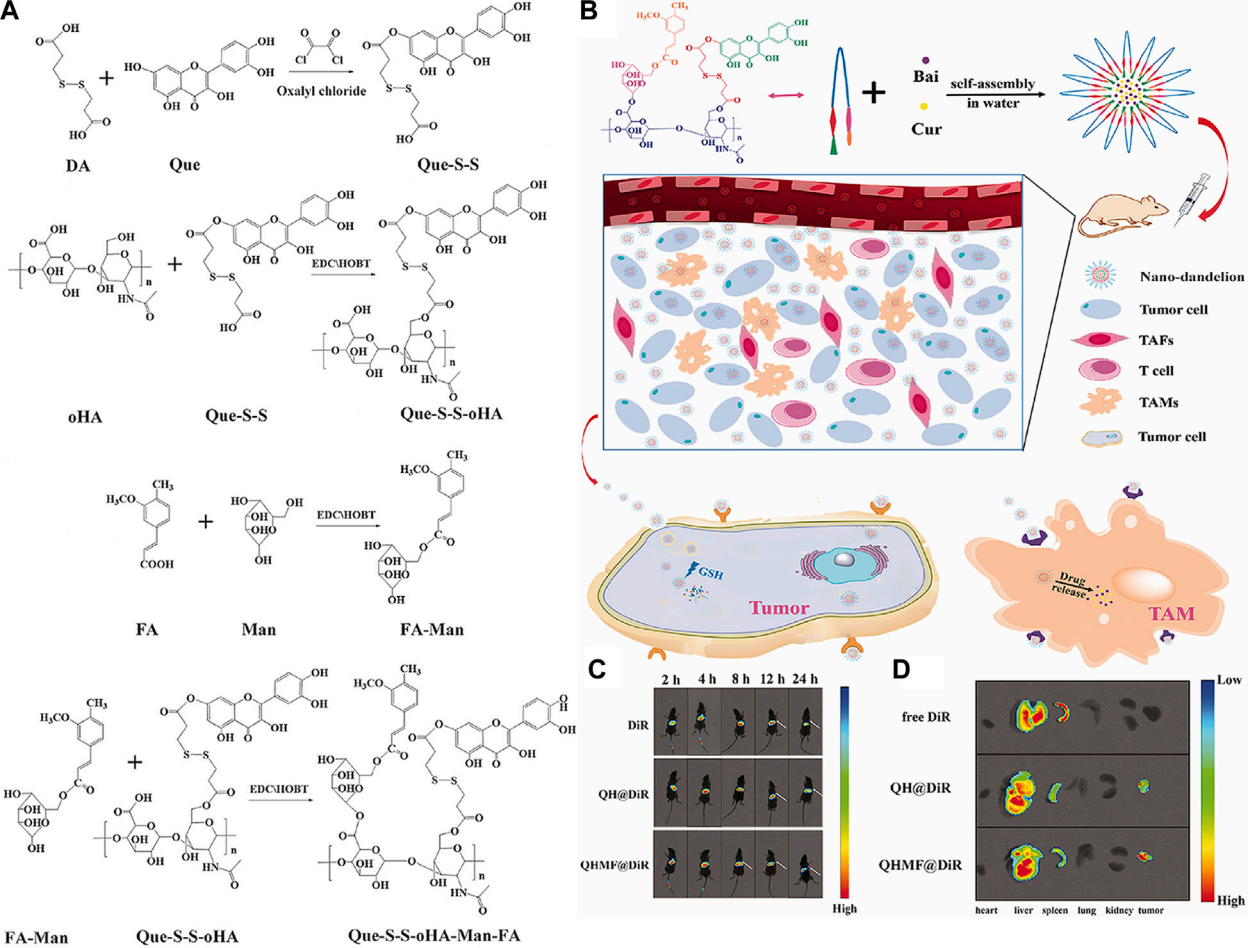
**(A)** Synthesis of QHMF. **(B)** Synthesis process of nano-dandelion and schematic diagram of nano-dandelion targeting A549 cells and TAMs. **(C)** The *in vivo* fluorescence imaging of A549 tumor-bearing mice was conducted at various time intervals (2, 4, 8, 12, and 24 h) subsequent to the intravenous injection of free DiR, QH@DiR, and QHMF@DiR. (Dir: Near-infrared fluorescent dye) **(D)** Distribution of free DiR, QH@DiR, and QHMF@DiR in the heart, liver, spleen, lung, kidney, and tumor.

Curcumin (Cur) (Figure 9A) is extracted from the rhizome of Curcuma longa, which contains two *O*-methylphenols and one *β*-Polyphenol compounds of diketones. Curcumin can regulate various pathways (NF-*k*B and PI3K/AKT signaling pathways, etc.) to induce tumor cell apoptosis and improve its sensitivity to radiotherapy and chemotherapy (Wang L. et al., 2019). Baicalin (Bai), which is shown in Figure 9B, is a flavonoid that originates from the root of Scutellaria baicalensis Georgi. Its capacity to target multiple signal pathways enables it to inhibit various forms of cancer (Singh et al., 2021). Wang et al. (2019a) synthesized quercetin-dithiodipropionic acid-oligomeric hyaluronic acid-mannose-ferulic acid (QHMF), prepared self-assembled targeting nano micelles (nano Taraxacum) (Figure 10A), which can simultaneously target to the tumor tissue. In addition, Taraxacum nanoparticles targeting CD44 receptors would recruit more nanoparticles at tumor sites. In addition, Man could specifically bind to CD206, making the nanoparticle more susceptible to tumor-associated macrophage (TAM) engulfment. The disulfide bond connected the hydrophobic part and the hydrophilic part, which can be destroyed by the excessive concentration of GSH in the tumor microenvironment (TME), thus releasing Cur/Bai (Figure 10B). The particle size of nanometer Taraxacum was 121.0 ± 15 nm, and the Zeta potential was −20.33 ± 4.02 mV. It was easy to enter tumor tissue and could be released for a long time (Figures 10C, D). Nanotaxacum has good cell penetration and tumor cytotoxicity with fewer side effects.

**FIGURE 11:**
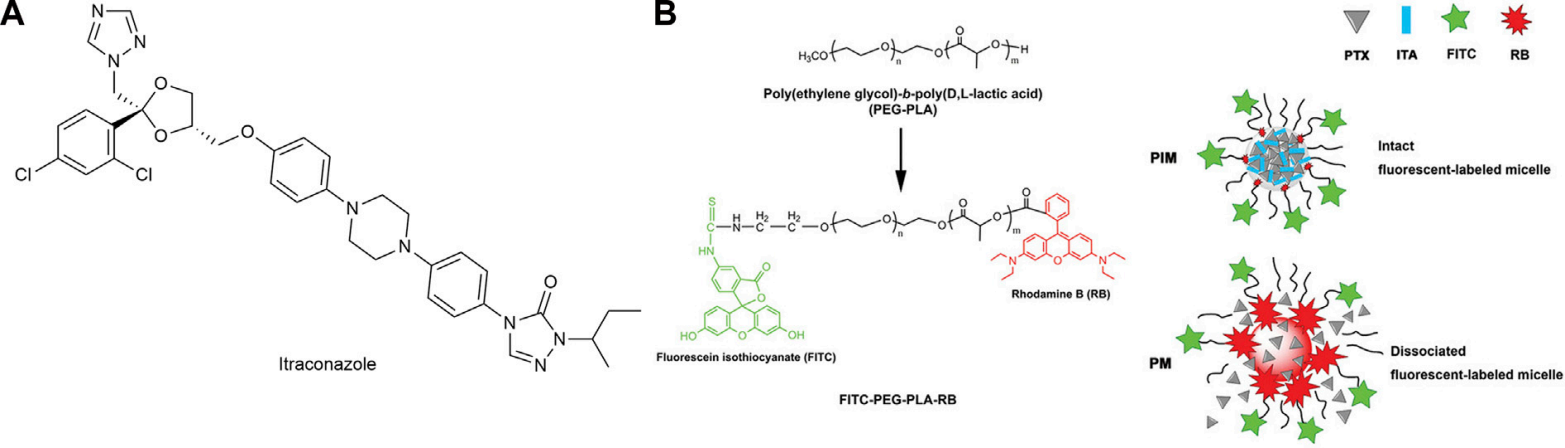
**(A)** Structure of Itraconazole. **(B)** Construction of fluorescent-labeled PEG-PLA and PEG-PLA micelles.

Itraconazole (ITA) (Figure 11A) is a widely used antifungal drug with strong anti-angiogenic activity, which can affect multiple angiogenic pathways (Aftab et al., 2011). Zhang et al. (2018) co-encapsulated PTX and ITA in PEG-PLA (poly lactic acid) micelles (PIM) (Figure 11B). There were strong intermolecular interactions between ITA and PTX, so the micelle stability was high and the critical micelle concentration was low. It was concluded from *in vitro* cell experiments that PIM enters cells mainly through endocytosis and could bypass the elevated efflux caused by P-glycoprotein over-expression. *In vitro* experiments showed that PIM was effective in significantly inhibiting PTX-resistant NSCLC. Meanwhile, ITA acted as a P-glycoprotein inhibitor, blocking P-glycoprotein-mediated efflux and increasing the cellular uptake of PTX. In xenografts obtained from patients with Kras mutation, orthotopic models, and subcutaneous models of paclitaxel resistance, PIM was found to significantly suppress tumor growth.

#### 3.2.6 Membrane-nanoparticle composites

Biological nanocarriers sourced from bacteria, viruses, and mammalian cells have become a popular area of research due to their biodegradable nature and specific functions in the body. They possess the ability to evade immune system attacks, prolong circulation time, and enhance selective targeting, making them an attractive option for various applications (Yoo et al., 2011). Biofilms also have the function of biological self-recognition and signal transduction. Based on these characteristics, membrane camouflage nanocarriers with good biocompatibility, targeting, and long clearance time were developed (Fang et al., 2014; Liu et al., 2022). Moreover, this method is easy to prepare with lower cost (Xu et al., 2023).

**FIGURE 12:**
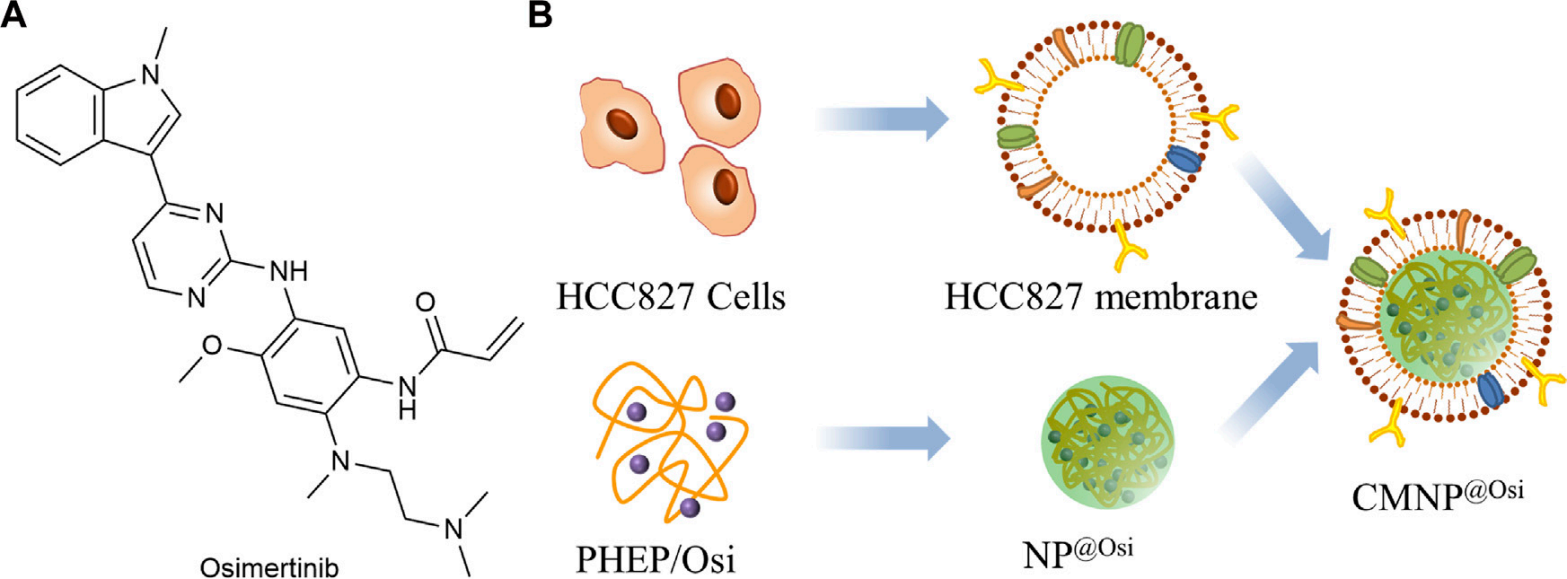
**(A)** Structure of Osimertinib. **(B)** Synthesis of CMNP^@Osi^.

As shown in Figure 12A, Osimertinib (Osi) is the third-generation EGFR-TKI used to treat patients with first or second generation acquired resistance to EGFR-TKI (Hognasbacka et al., 2023). Xu et al. (2023) prepared the inner core NP^@Osi^ nanoparticles by nanoprecipitation using polymeric polyphosphoester (PHEP) (Figure 12B). The cell membranes were extracted from HCC827 cells and fused with NP^@Osi^ to obtain CMNP^@Osi^. The average particle size was 100 nm, the IC50 value was 14.23 nM, while the release of Osi was not affected by the cell membrane. *In vivo* administration of HCC827 xenografted BALB/c nude mice confirmed that the nanoparticles had good anti-tumor activity and biocompatibility. Compared to other cell types, CMNP^@Osi^ exhibited better affinity and stronger targeting of its homologous cells. Therefore, homologous targeting can be considered as a specific targeting function of biofilms. At the same time, Osi, as a targeted drug, has been selectively used for EFGR sensitivity mutations and T790M resistance mutations. CMNP^@Osi^ implemented the dual targeting strategy and facilitated the application of the membrane coating technique.

**FIGURE 13:**
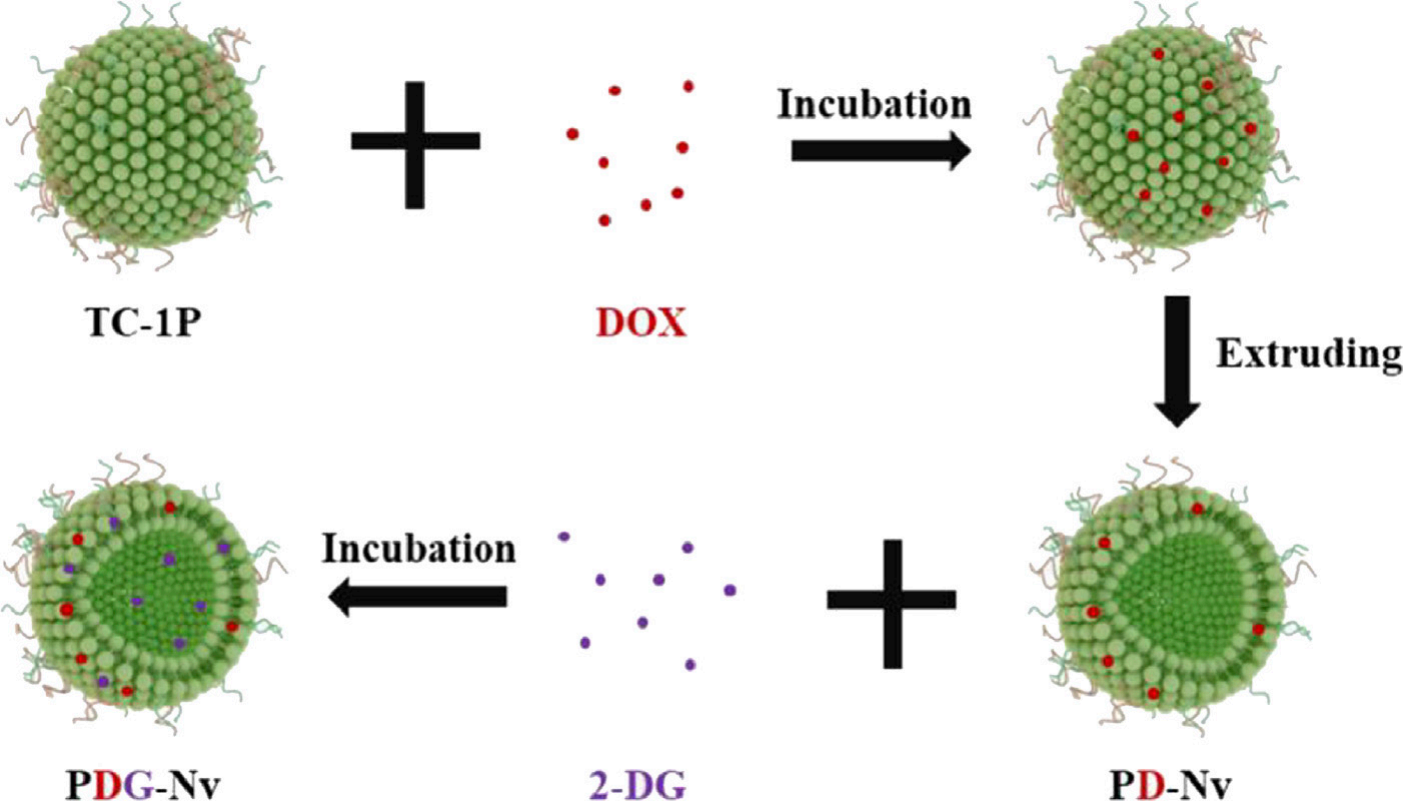
The assembly process of DOX and 2-DG loaded P-NV.

Over the past few years, the research for immune checkpoint blocking (ICB) drugs has progressed significantly with the development of inhibitors targeting programmed cell death protein 1 (PD-1) and its associated ligand, PD-L1. Although the use of antibody inhibitors targeting PD-1 or PD-L1 has shown effectiveness in the treatment of lung cancer, they are unfortunately only effective in 10%–30% of patients (Li B. et al., 2023). Li et al. (2023a) designed TC1 cells (mouse NSCLC cell line), prepared PD-1 nanovesicles (P-NVs) by extrusion method, and loaded DOX and 2-deoxy-D-glucose (2-DG) into P-NVs to obtain PDG-NV for targeted treatment of NSCLC (Figure 13). The enhancement of tumor targeting capability was achieved by incorporating PD-1 onto NVs. Moreover, the combination of efficient loading of 2-DG and DOX exhibited a synergistic therapeutic effect on NSCLC in both *in vitro* and *in vivo* experiments, leading to significant cytotoxicity on tumor cells. PDG-NVs were also found to effectively reduce PD-L1 expression in the TME and improve the antitumor immune response mediated by CD4^+^ and CD8^+^ T cells. Thus, PDG-NV had great therapeutic potential, but this needs to be confirmed by further clinical trials.

Cisplatin (CDDP) is a platinum complex and is considered one of the most efficacious chemotherapy drugs for cancer treatment (Wang and Lippard, 2005). Despite its considerable anticancer activity, cisplatin’s clinical application is restricted because of its acquired resistance and severe side effects (Yimit et al., 2019). Liang et al. (2021) used tannic acid (TA) to chelate Fe-rich metal-organic framework nanoparticles (MOF) to create MOF/TA. To create MOF/TA-CDDP-APT (MTCA), they further equipped the MOF/TA with CDDP and utilized antagonistic DNA aptamers (Apt) that specifically target PD-L1. The MTCA was further enveloped in the cancer cell membrane to create M@MTCA. The complex delivered the contents precisely to the tumor under the acidic conditions of TME, then released therapeutic substances and iron ions (Fe^2+^) reactively with degradation. In addition to chemotherapy, CDDP can also be used to treat NSCLC through chemodynamic therapy. The level of H_2_O_2_ in the cell was raised by cascade reaction, and H_2_O_2_ was continuously converted to high cytotoxic •OH by Fenton reaction. Moreover, the nanocomplex was capable of converting “cold” tumors into “hot” tumors by promoting immunogenic cell death in tumor cells, which can enhance the therapeutic effectiveness when combined with ICBs (Apt).

**FIGURE 14:**
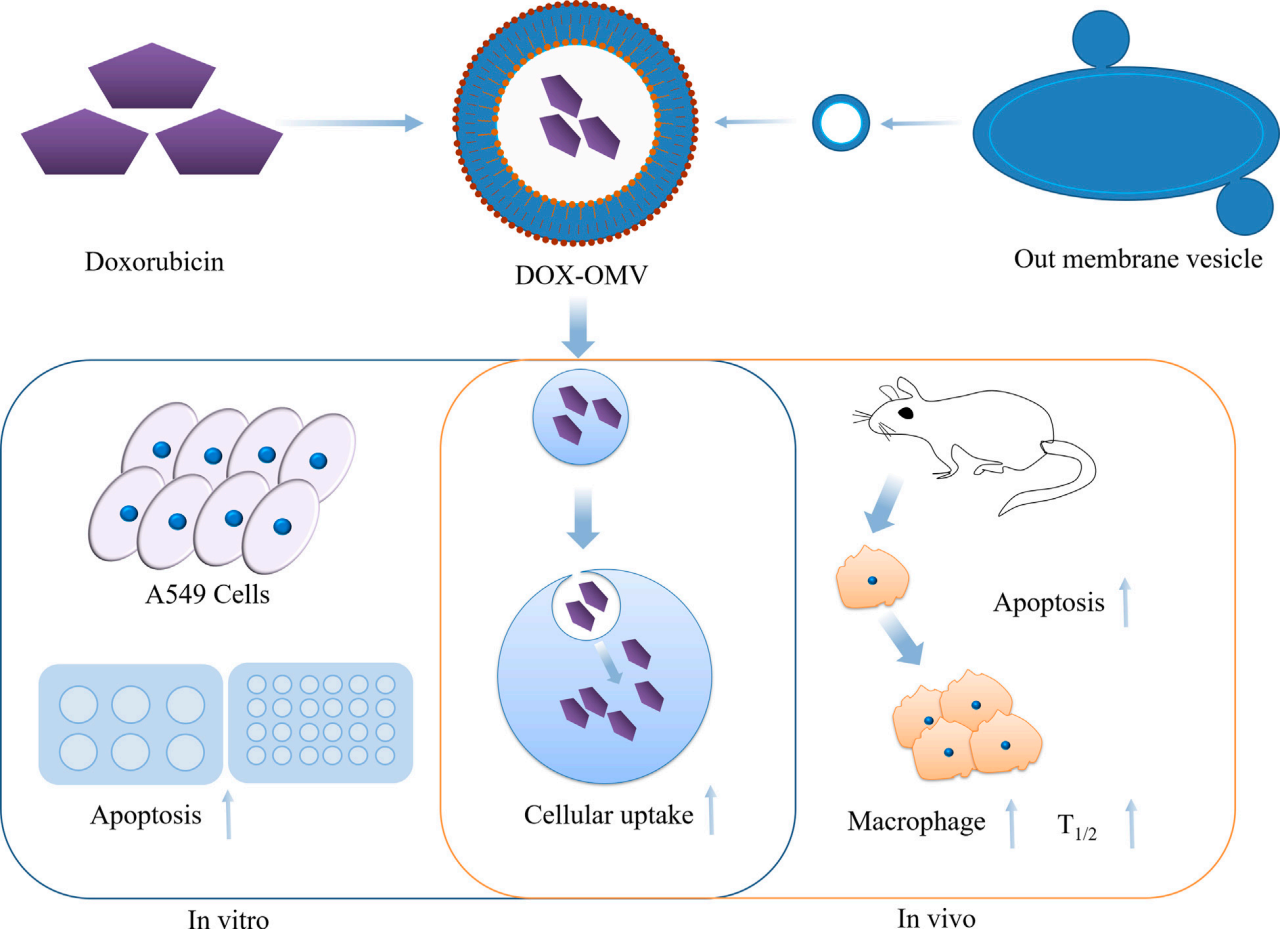
Overview of the anti-NSCLC effects of DOX-OMV. *In vitro*, DOX in OMV is delivered into A549 cells, resulting in strong cytotoxicity and apoptosis. *In vivo*, OMV also induces the recruitment of macrophages in tumor microwells.

Outer membrane vesicles (OMVs) are phospholipid bilayer structures with a spherical shape, ranging in size from 20 to 250 nm. These vesicles are generated from the outer membrane and periplasm of Gram-negative bacteria. They are naturally released from the bacterial cell envelope during their growth process (Schwechheimer and Kuehn, 2015). OMVs are engaged in a diverse array of physiological activities, such as intracellular and extracellular communication, quorum sensing, and stress responses. Kuerban et al. (2020) obtained OMV from attenuated *Klebsiella pneumoniae* and loaded DOX onto them (DOX-OMV) (Figure 14). The results of confocal microscopy and *in vivo* distribution studies indicated that the DOX-OMV delivery system effectively delivered DOX to NSCLC A549 cells. DOX-OMV also demonstrated a powerful cytotoxic effect, resulting in the triggering of cancerous cell death. When tested on BALB/c nude mice with A549 tumors, DOX-OMV was observed to significantly inhibit tumor growth, while being well-tolerated and exhibiting desirable pharmacokinetic properties. Moreover, OMVs are immunogenic and can recruit macrophages in TME causing corresponding immune responses, so they have great potential in tumor chemical immunotherapy.

#### 3.2.7 Microspheres

Recently, the importance of long-acting implants has been increasingly recognized due to their unique advantages such as fewer medication administrations, high patient compliance, and low toxicity and side effects (Qiu et al., 2022). Biodegradable polymer microspheres possess the aforementioned advantages. These microspheres not only exhibit excellent biocompatibility but can also be prepared as sustained or controlled release formulations according to needs. Furthermore, their particle size distribution is narrow, eliminating the need for screening of improperly sized microspheres in later stages. As a result, this characteristic reduces raw material waste and lowers preparation costs. Additionally, microspheres demonstrate good batch-to-batch reproducibility and repeatable release behavior, showcasing significant clinical value (Jin et al., 2020). Microsphere formulations belong to the high-end and complex field of pharmaceutical development, with a higher threshold. There is relatively little homogeneous competition in the pharmaceutical market for such formulations. For most drugs, there is a significant market capacity and room for growth, making their commercial prospects vast.

Poly lactic-co-glycolic acid (PLGA) formulations consist of different types, such as microspheres, *in situ* gel systems, and solid implants, and are commonly utilized for controlled and sustained drug release (Li X. et al., 2022). So far, there are more than twenty PLGA microsphere-based commercial products accessible in the market, including Decapeptyl Depot (Anwar et al., 2023), Lupron Depot, and Eligard (Kapoor et al., 2015). Sorafenib is a receptor tyrosine kinase inhibitor that can be taken orally, and it targets multiple receptors. The drug is capable of suppressing the proliferation of tumor cells, hindering angiogenesis, and promoting apoptosis of tumor cells. Sorafenib is primarily utilized to treat advanced renal cell carcinoma. Li et al. (2020) used a double-emulsion solvent diffusion method to encapsulate sorafenib (SOR) and catalase (CAT) into PLGA microspheres, forming SOR-CAT-PLGA microspheres (SOR-CAT-PLGA MSs). The size of these microspheres is approximately 73.5 ± 8.3 μm, with encapsulation efficiencies of 68.7% ± 1.9% for SOR and 76.5% ± 2.3% for CAT. The combined application of SOR and CAT demonstrates a significant synergistic effect in promoting cancer cell apoptosis while causing minimal damage to surrounding normal cells. During *in vivo* experiments, SOR-CAT-PLGA microspheres were found to fill tumor blood vessels effectively, impede neovascularization, and result in the shrinkage of tumor volume or prompt necrosis in rabbits. Moreover, these microspheres also downregulate the expression of PD-L1, suppress CD8^+^ T cell apoptosis, and elevate IFN-γ levels. Therefore, the combined use of SOR and CAT may be an effective treatment method for liver cancer.

Porous microspheres (pMS) possess a large specific surface area and excellent adsorption performance, with interconnected inner and outer channels. pMS have broad application prospects in gastric retention drug delivery, pulmonary targeting drug delivery, high-speed chromatography, tissue regeneration scaffold development, and serving as carriers for biopharmaceuticals (Ghosh Dastidar et al., 2018). Due to the low density and light weight of pMS, Yang et al. (2019) designed a dry powder inhalation drug delivery system based on pMS for treating EGFR TKI-resistant NSCLC. They loaded AFT lipid nanoparticles (AFT-SLN) and PTX into PLGA-pMS, creating AFT-SLN-PTX-pMS. This microsphere complex exhibited two distinct drug release phases, with PTX releasing faster than AFT. The aerodynamic properties of AFT-SLN-PTX-pMS were found to be favorable, with an MMAD value of about 3.25 µm, indicating successful deposition in the alveolar region of the lungs. According to pharmacokinetic parameters, both drugs maintained high concentrations within 96 h and were predominantly concentrated in the lungs. Additionally, the combination of the two drugs showed synergistic effects, with PTX significantly enhancing the cancer cell growth inhibitory effect of AFT. Moreover, AFT’s sustained release inhibited cancer cell migration. These results suggest that AFT-SLN-PTX-pMS can evade phagocytic cells and achieve prolonged retention in the lungs. This drug delivery system demonstrates passive lung targeting characteristics, enhancing tumor killing efficacy while reducing systemic side effects.

Tumor treatment is a long-term process, and direct injection of drugs into the tumor presents a promising approach. Some drugs have been attempted to be administered directly into tumor tissues. However, these drugs have poor retention at the administration site and may leak out of the tumor within a few hours. This significantly affects drug efficacy and may increase systemic toxicity of the medication (Nakajima et al., 2000; Wang et al., 2003; Muñoz et al., 2021). Drug depot technology may be a potential approach to address these issues. Drugs diffuse and gradually release within a polymer matrix, (such as PLA or PLGA). However, the stability of sensitive drugs may be impacted by the properties of the polymer matrix, including its hydrophobic nature, internal pH, and reactive functional groups (Lessmann et al., 2023). Thermosensitive hydrogel is a drug delivery system whose physical state changes with temperature. By enhancing the adhesiveness of drugs to the affected area and prolonging local residence time, thermosensitive hydrogels can effectively improve drug bioavailability (Fan et al., 2022). Gel solutions undergo a phase transition with changes in environmental temperature and quickly solidify to block blood vessels, thereby cutting off the blood supply to tumors. Additionally, it can effectively address issues of embolism caused by non-uniform sizes of lipids and microspheres. Compared to common tumor treatment approaches, thermosensitive hydrogels can serve as drug depots for controlled release of drugs and targeted delivery into tumor tissues. The irregular shape of the affected area in tumor treatment poses challenges for drug administration, making thermosensitive hydrogels an ideal drug carrier (Ma and Yan, 2021).

Yu et al. (2019) utilized a modified double emulsion-solvent evaporation technique to fabricate microspheres of losartan potassium (LP MSs), with gelatin serving as the inner phase. These microspheres were then combined with cisplatin-loaded nanoparticles (CDDP NPs) made of poly (*α*-*L*-glutamate) grafted polyethylene glycol mono methyl ether (PLG-gmPEG). The microspheres and CDDP NPs were then dissolved in a temperature-responsive hydrogel, which could prevent drug leakage from the needle hole and greatly improve the drug retention time in the tumor. In *in vivo* experiments, LP MSs/CDDP NPs gel was found to effectively suppress tumor growth, decrease tumor volume, and caused no damage to the surrounding muscle tissue. This microsphere-gel composite has practical significance for the treatment of solid tumors. Tan et al. (2021) developed an injectable platform (Cur-MP/IR820 gel) for the treatment of osteosarcoma and the regeneration of bone tissue, which consisted of a composite methylcellulose hydrogel containing Cur microspheres and IR820. In *in vitro* experiments, after laser irradiation and heating, Cur was released rapidly, and the cytotoxicity against tumor cells was significantly higher than in other groups. Then, the mice in the group that received laser irradiation and were given Cur-MP/IR820 experienced high temperatures (approximately 51°C) at the tumor site, leading to tumor elimination. Subsequently, the platform continued to release Cur, killing the remaining cancer cells and promoting the differentiation of mesenchymal stem cells into osteoblasts and calcium deposition. The Cur-MP/IR820 hydrogel platform offers a promising avenue for the treatment of bone tumors and the regeneration of bone tissue, thereby providing a source of inspiration for future research in this area.

## 4 Conclusion

The development of nanotechnology has provided an excellent platform for tumor diagnosis, tumor cell imaging, early prevention and targeted therapy. The introduction of nanomaterials has provided novel functional technologies for the early diagnosis and treatment of major diseases such as cancer. In this paper, we have summarized the progress of nanotechnology in the early diagnosis of tumors, including the development status of nanomaterials for *in vivo* imaging and *in vitro* imaging techniques. The challenges and future directions of nanoprobe technology in image-guided oncology diagnostics are discussed, as well as the prospects for the development of biosensors for high sensitivity detection of tumor biomarkers. They have their advantages, such as magnetic nanoparticles that can be used as medical contrast agents for increased sensitivity, nanocrystalline QDs with high stability, and gold nanoparticles with low toxicity. These nanoparticles enable ultra-fast, highly sensitive and highly selective detection and diagnosis. At the same time, however, these tumor detection techniques suffer from several drawbacks, such as the large amount of residual nanoprobes in the body, and the possibility of cell death, oxidative stress and inflammation, among other side effects, due to too long residence time.

Conventional cancer therapies, targeted therapies and immunotherapies all come with their own set of limitations, including resistance, systemic toxicity and rapid elimination rates. New modalities of cancer treatment may be combined with nanotechnology to alter the status of treatment with excessive systemic chemotherapy. There is a growing clinical focus on the application of nanotechnology with targeted and slow-release properties to conventional oncology treatments, with nanomedicine being the leading candidate.

Nanomedicines for the treatment of tumors are generally natural nanomedicines, polymeric nanodrug carriers, and nanoliposomes. The emergence of emerging Nab-P, mRNA-LNP, protein nanocages, micelles, membrane-nanoparticle composites and microspheres has solved many problems in clinical cancer prevention and treatment. Nevertheless, there exist certain obstacles in the practical implementation of these nanomedicines in clinical settings. The instability of the phospholipid component of liposomal drugs has hampered the translation of liposomal research into clinical practice for more than 50 years. The emergence of mRNA-LNP has represented a significant advancement in cancer prevention, yet there is still ample scope for enhancing LNP effectiveness. This can be accomplished through the refinement of lipid composition and customizing LNPs to cater to specific objectives. Cell membrane-coated biomimetic nanoparticles have shown great potential for cancer targeted therapies due to their superior molecular identification specificity and cancer cell targeting. However, there is still a lack of key indicators that can assess the quality of these bio-inspired nanoparticles and the resulting therapeutic effects. Additionally, only a few of these nanoparticles have received clinical approval due to their toxicity, immunogenicity and reticuloendothelial system isolation. Microsphere formulations are a type of sustained-release drug delivery system that can stably release drugs over a prolonged period. They are mainly used for diseases that require frequent dosing, such as cancer and psychiatric disorders. This drug delivery system can significantly improve the bioavailability of drugs and patient compliance. However, the development and production of microsphere formulations are challenging, and there are high barriers to entry in the industry. Therefore, it is necessary to accelerate the development of corresponding technologies. There is no denying that nanotechnology will play an important role in the future of medicine, but there are many aspects of nanotechnology that are not yet perfect. For nanotechnology to benefit humanity, further exploration by relevant researchers is needed.

## References

[B1] Aftab B. T. Dobromilskaya I. Liu J. O. Rudin C. M. (2011). Itraconazole inhibits angiogenesis and tumor growth in non-small cell lung cancer. Cancer Res. 71 (21), 6764–6772. 10.1158/0008-5472.CAN-11-0691 21896639 PMC3206167

[B2] Andresen T. L. Larsen J. B. (2020). Compositional inhomogeneity of drug delivery liposomes quantified at the single liposome level. Acta Biomater. 118, 207–214. 10.1016/j.actbio.2020.10.003 33065286

[B3] Anwar A. Sun P. Rong X. Arkin A. Elham A. Yalkun Z. (2023). Process analytical technology as in-process control tool in semi-continuous manufacturing of PLGA/PEG-PLGA microspheres. Heliyon 9 (5), e15753. 10.1016/j.heliyon.2023.e15753 37153380 PMC10160502

[B4] Baysal H. De Pauw I. Zaryouh H. De Waele J. Peeters M. Pauwels P. (2020). Cetuximab-induced natural killer cell cytotoxicity in head and neck squamous cell carcinoma cell lines: Investigation of the role of cetuximab sensitivity and HPV status. Br. J. Cancer 123 (5), 752–761. 10.1038/s41416-020-0934-3 32541873 PMC7462851

[B5] Bevers S. Kooijmans S. A. A. Van de Velde E. Evers M. J. W. Seghers S. Gitz-Francois J. J. J. M. (2022). mRNA-LNP vaccines tuned for systemic immunization induce strong antitumor immunity by engaging splenic immune cells. Mol. Ther. 30 (9), 3078–3094. 10.1016/j.ymthe.2022.07.007 35821637 PMC9273295

[B6] Chakravarty K. Dalal D. C. (2018). Mathematical modelling of liposomal drug release to tumour. Math. Biosci. 306, 82–96. 10.1016/j.mbs.2018.10.012 30391313

[B7] Chatterjee P. Kumar S. (2022). Current developments in nanotechnology for cancer treatment. Mater. Today Proc. 48, 1754–1758. 10.1016/j.matpr.2021.10.048

[B8] Chen F. Wang M. Du Z. Pu X. Zhu B. (2023). 131I labeled pH-responsive gold nanoparticles for bimodal tumor diagnosis. Mater. Lett. 330, 133202. 10.1016/j.matlet.2022.133202

[B9] Cheng Y. Ling S. D. Geng Y. Wang Y. Xu J. (2021). Microfluidic synthesis of quantum dots and their applications in bio-sensing and bio-imaging. Nanoscale Adv. 3 (8), 2180–2195. 10.1039/d0na00933d 36133767 PMC9417800

[B10] de Oliveira Silva J. Fernandes R. S. Ramos Oda C. M. Ferreira T. H. Machado Botelho A. F. Martins Melo M. (2019). Folate-coated, long-circulating and pH-sensitive liposomes enhance doxorubicin antitumor effect in a breast cancer animal model. Biomed. Pharmacother. 118, 109323. 10.1016/j.biopha.2019.109323 31400669 PMC7104811

[B11] Eivazzadeh-Keihan R. Bahreinizad H. Amiri Z. Aliabadi H. A. M. Salimi-Bani M. Nakisa A. (2021). Functionalized magnetic nanoparticles for the separation and purification of proteins and peptides. TrAC Trends Anal. Chem. 141, 116291. 10.1016/j.trac.2021.116291

[B12] Fan R. Liu Y. Zhang T. Wang Z. Zhang H. Li J. (2022). Based on clinical application research progress of thermosensitive gel in different drug delivery sites. Acta Pharm. Sin. 57 (05), 1235–1244. 10.16438/j.0513-4870.2021-1209

[B13] Fang R. H. Hu C. M. Luk B. T. Gao W. Copp J. A. Tai Y. (2014). Cancer cell membrane-coated nanoparticles for anticancer vaccination and drug delivery. Nano Lett. 14 (4), 2181–2188. 10.1021/nl500618u 24673373 PMC3985711

[B14] Ganesh S. Venkatakrishnan K. Tan B. (2022). Early detection and prediction of cancer metastasis – unravelling metastasis initiating cell as a dynamic marker using self-functionalized nanosensors. Sensors Actuators B Chem. 361, 131655. 10.1016/j.snb.2022.131655

[B15] Ghosh B. Biswas S. (2021). Polymeric micelles in cancer therapy: State of the art. J. Control Release 332, 127–147. 10.1016/j.jconrel.2021.02.016 33609621

[B16] Ghosh Dastidar D. Saha S. Chowdhury M. (2018). Porous microspheres: Synthesis, characterisation and applications in pharmaceutical & medical fields. Int. J. Pharm. 548 (1), 34–48. 10.1016/j.ijpharm.2018.06.015 29940297

[B17] Goswami S. Chiang C.-L. Zapolnik K. Nunes J. Ventura A. Mo X. (2022). ROR1 targeted immunoliposomal delivery of OSU-2S shows selective cytotoxicity in t(1;19)(q23;p13) translocated B-cell acute lymphoblastic leukemia. Leukemia Res. 118, 106872. 10.1016/j.leukres.2022.106872 35640397 PMC10029232

[B18] Granados-Riveron J. T. Aquino-Jarquin G. (2021). Engineering of the current nucleoside-modified mRNA-LNP vaccines against SARS-CoV-2. Biomed. Pharmacother. 142, 111953. 10.1016/j.biopha.2021.111953 34343897 PMC8299225

[B19] Granja A. Lima-Sousa R. Alves C. G. de Melo-Diogo D. Nunes C. Sousa C. T. (2023). Multifunctional targeted solid lipid nanoparticles for combined photothermal therapy and chemotherapy of breast cancer. Biomater. Adv. 151, 213443. 10.1016/j.bioadv.2023.213443 37146526

[B20] Gulia M. Nishal S. Maddiboyina B. Dutt R. Kumar Desu P. Wadhwa R. (2023). Physiological pathway, diagnosis and nanotechnology based treatment strategies for ovarian cancer: A review. Med. Omics 8, 100020. 10.1016/j.meomic.2023.100020

[B21] Halevas E. Mavroidi B. Swanson C. H. Smith G. C. Moschona A. Hadjispyrou S. (2019). Magnetic cationic liposomal nanocarriers for the efficient drug delivery of a curcumin-based vanadium complex with anticancer potential. J. Inorg. Biochem. 199, 110778. 10.1016/j.jinorgbio.2019.110778 31442839

[B22] He K. Tang M. (2018). Safety of novel liposomal drugs for cancer treatment: Advances and prospects. Chemico-Biological Interact. 295, 13–19. 10.1016/j.cbi.2017.09.006 28919304

[B23] Hognasbacka A. Poot A. J. Kooijman E. Schuit R. C. Schreurs M. Verlaan M. (2023). Synthesis and preclinical evaluation of two osimertinib isotopologues labeled with carbon-11 as PET tracers targeting the tyrosine kinase domain of the epidermal growth factor receptor. Nucl. Med. Biol. 120-121, 108349. 10.1016/j.nucmedbio.2023.108349 37209556

[B24] Hu Y. Zhang J. Hu H. Xu S. Xu L. Chen E. (2020). Gefitinib encapsulation based on nano-liposomes for enhancing the curative effect of lung cancer. Cell Cycle 19 (24), 3581–3594. 10.1080/15384101.2020.1852756 33300430 PMC7781631

[B25] Hu Z. Zhang R. Xu S. Wang J. Li X. Hu J. (2023). Construction of nano-drug delivery and antitumor system of stimuli-responsive polypeptides. Colloids Surfaces B Biointerfaces 226, 113310. 10.1016/j.colsurfb.2023.113310 37054465

[B26] Huang C. Dong H. Su Y. Wu Y. Narron R. Yong Q. (2019). Synthesis of carbon quantum dot nanoparticles derived from byproducts in bio-refinery process for cell imaging and *in vivo* bioimaging. Nanomater. (Basel) 9 (3), 387. 10.3390/nano9030387 PMC6473984 30866423

[B27] Jha A. Viswanadh M. K. Burande A. S. Mehata A. K. Poddar S. Yadav K. (2020). DNA biodots based targeted theranostic nanomedicine for the imaging and treatment of non-small cell lung cancer. Int. J. Biol. Macromol. 150, 413–425. 10.1016/j.ijbiomac.2020.02.075 32057849

[B28] Jin H. Chong H. Zhu Y. Zhang M. Li X. Bazybek N. (2020). Preparation and evaluation of amphipathic lipopeptide-loaded PLGA microspheres as sustained-release system for AIDS prevention. Eng. Life Sci. 20 (11), 476–484. 10.1002/elsc.202000026 33204234 PMC7645643

[B29] Kapoor D. N. Bhatia A. Kaur R. Sharma R. Kaur G. Dhawan S. (2015). Plga: A unique polymer for drug delivery. Ther. Deliv. 6 (1), 41–58. 10.4155/tde.14.91 25565440

[B30] Karthikeyan L. Sobhana S. Yasothamani V. Gowsalya K. Vivek R. (2023). Multifunctional theranostic nanomedicines for cancer treatment: Recent progress and challenges. Biomed. Eng. Adv. 5, 100082. 10.1016/j.bea.2023.100082

[B31] Khafoor A. A. Karim A. S. Sajadi S. M. (2023). Recent progress in synthesis of nano based liposomal drug delivery systems: A glance to their medicinal applications. Results Surfaces Interfaces 11, 100124. 10.1016/j.rsurfi.2023.100124

[B32] Khan M. I. Batool F. Ali R. Zahra Q. u. A. Wang W. Li S. (2022). Tailoring radiotherapies and nanotechnology for targeted treatment of solid tumors. Coord. Chem. Rev. 472, 214757. 10.1016/j.ccr.2022.214757

[B33] Koyama N. Watanabe Y. Iwai Y. Miwa C. Nagai Y. Aoshiba K. (2018). Effectiveness of nanoparticle albumin-bound paclitaxel plus carboplatin in non-small lung cancer patients with malignant pleural effusion. Neoplasma 65 (1), 132–139. 10.4149/neo_2018_170206N78 29322797

[B34] Kuerban K. Gao X. Zhang H. Liu J. Dong M. Wu L. (2020). Doxorubicin-loaded bacterial outer-membrane vesicles exert enhanced anti-tumor efficacy in non-small-cell lung cancer. Acta Pharm. Sin. B 10 (8), 1534–1548. 10.1016/j.apsb.2020.02.002 32963948 PMC7488491

[B35] Kundranda M. N. Niu J. (2015). Albumin-bound paclitaxel in solid tumors: Clinical development and future directions. Drug Des. Dev. Ther. 9, 3767–3777. 10.2147/DDDT.S88023 PMC4521678 26244011

[B36] Kuznetsov D. Dezhurov S. Krylsky D. Neschisliaev V. (2022). Fluorescent nanosensors for molecular visualization of the c-Met tumor marker. Nano-Structures Nano-Objects 31, 100890. 10.1016/j.nanoso.2022.100890

[B37] Lai K. C. Chueh F. S. Hsiao Y. T. Cheng Z. Y. Lien J. C. Liu K. C. (2019). Gefitinib and curcumin-loaded nanoparticles enhance cell apoptosis in human oral cancer SAS cells *in vitro* and inhibit SAS cell xenografted tumor *in vivo* . Toxicol. Appl. Pharmacol. 382, 114734. 10.1016/j.taap.2019.114734 31470033

[B38] Le T. T. Wu M. Lee J. H. Bhatt N. Inman J. T. Berger J. M. (2023). Etoposide promotes DNA loop trapping and barrier formation by topoisomerase II. Nat. Chem. Biol. 19 (5), 641–650. 10.1038/s41589-022-01235-9 36717711 PMC10154222

[B39] Lee W. H. Loo C. Y. Ghadiri M. Leong C. R. Young P. M. Traini D. (2018). The potential to treat lung cancer via inhalation of repurposed drugs. Adv. Drug Deliv. Rev. 133, 107–130. 10.1016/j.addr.2018.08.012 30189271

[B40] Lessmann T. Jones S. A. Voigt T. Weisbrod S. Kracker O. Winter S. (2023). Degradable hydrogel for sustained localized delivery of anti-tumor drugs. J. Pharm. Sci. S0022-3549, 00227-7. 10.1016/j.xphs.2023.05.018 37279836

[B41] Li B. Yang T. Liu J. Yu X. Li X. Qin F. (2023a). Genetically engineered PD-1 displaying nanovesicles for synergistic checkpoint blockades and chemo-metabolic therapy against non-small cell lung cancer. Acta Biomater. 161, 184–200. 10.1016/j.actbio.2023.03.002 36893957

[B42] Li H. Lin L. Yan R. Chen Z. Wen X. Zeng X. (2022a). Multi-functional Fe3O4@HMPDA@G5-Au core-releasable satellite nano drug carriers for multimodal treatment of tumor cells. Eur. Polym. J. 181, 111647. 10.1016/j.eurpolymj.2022.111647

[B43] Li J. Lou K. Dang Y. Tian H. Luo Q. Huang C. (2023b). Precise tumor resection under the navigation of Tumor-Microenvironment pH-activated NIR-II fluorescence imaging via calcium Carbonate/Polydopamine Co-packed Nd-doped downshifting nanoprobes. Mater. Des. 227, 111703. 10.1016/j.matdes.2023.111703

[B44] Li J. Zhu L. Kwok H. F. (2023c). Nanotechnology-based approaches overcome lung cancer drug resistance through diagnosis and treatment. Drug Resist. Updat. 66, 100904. 10.1016/j.drup.2022.100904 36462375

[B45] Li W. Jing Z. Wang S. Li Q. Xing Y. Shi H. (2021). P22 virus-like particles as an effective antigen delivery nanoplatform for cancer immunotherapy. Biomaterials 271, 120726. 10.1016/j.biomaterials.2021.120726 33636548

[B46] Li X. Qin J. Hu Y. (2023d). Switch-on hydrogel biosensor based on self-assembly Mn-doped ZnS QDs and cellulose nanofibrils for glutathione detection. Microchem. J. 191, 108763. 10.1016/j.microc.2023.108763

[B47] Li X. Yu H. Huang Y. Chen Y. Wang J. Xu L. (2020). Preparation of microspheres encapsulating sorafenib and catalase and their application in rabbit VX2 liver tumor. Biomed. Pharmacother. 129, 110512. 10.1016/j.biopha.2020.110512 32768982

[B48] Li X. Zhang Z. Harris A. Yang L. (2022b). Bridging the gap between fundamental research and product development of long acting injectable PLGA microspheres. Expert Opin. Drug Deliv. 19 (10), 1247–1264. 10.1080/17425247.2022.2105317 35863759

[B49] Liang L. Wen L. Weng Y. Song J. Li H. Zhang Y. (2021). Homologous-targeted and tumor microenvironment-activated hydroxyl radical nanogenerator for enhanced chemoimmunotherapy of non-small cell lung cancer. Chem. Eng. J. 425, 131451. 10.1016/j.cej.2021.131451

[B50] Liu H. Miao Z. Zha Z. (2022). Cell membrane-coated nanoparticles for immunotherapy. Chin. Chem. Lett. 33 (4), 1673–1680. 10.1016/j.cclet.2021.10.057

[B51] Liu J. Zhang J. Gao Y. Jiang Y. Guan Z. Xie Y. (2023). Barrier permeation and improved nanomedicine delivery in tumor microenvironments. Cancer Lett. 562, 216166. 10.1016/j.canlet.2023.216166 37028698

[B52] Lu Q. (2023). Bioresponsive and multifunctional cyclodextrin-based non-viral nanocomplexes in cancer therapy: Building foundations for gene and drug delivery, immunotherapy and bioimaging. Environ. Res. 234, 116507. 10.1016/j.envres.2023.116507 37364628

[B53] Lv W. Xu C. Wu H. Zhu Y. Akakuru O. U. Du H. (2022). Ultrasound-visualized nanocarriers with siRNA for targeted inhibition of M2-like TAM polarization to enhance photothermal therapy in NSCLC. Nano Res. 16 (1), 882–893. 10.1007/s12274-022-4767-7

[B54] Ma N. Yan Z. (2021). Research progress of thermosensitive hydrogel in tumor therapeutic. Nanoscale Res. Lett. 16 (1), 42. 10.1186/s11671-021-03502-5 33665739 PMC7933296

[B55] Ma Y. Cong Z. Gao P. Wang Y. (2023). Nanosuspensions technology as a master key for nature products drug delivery and *in vivo* fate. Eur. J. Pharm. Sci. 185, 106425. 10.1016/j.ejps.2023.106425 36934992

[B56] Mazieres J. Kowalski D. Luft A. Vicente D. Tafreshi A. Gümüş M. (2020). Health-related quality of life with carboplatin-paclitaxel or nab-paclitaxel with or without pembrolizumab in patients with metastatic squamous non-small-cell lung cancer. J. Clin. Oncol. 38 (3), 271–280. 10.1200/jco.19.01348 31751163

[B57] Miao H. Huang K. Li Y. Li R. Zhou X. Shi J. (2023). Optimization of formulation and atomization of lipid nanoparticles for the inhalation of mRNA. Int. J. Pharm. 640, 123050. 10.1016/j.ijpharm.2023.123050 37201764

[B58] Milovanovic M. Arsenijevic A. Milovanovic J. Kanjevac T. Arsenijevic N. (2017). “Nanoparticles in antiviral therapy,” in Antimicrobial nanoarchitectonics. (Elsevier), 383–410. 10.1016/B978-0-323-52733-0.00014-8

[B59] Montiel Schneider M. G. Lassalle V. L. (2017). Magnetic iron oxide nanoparticles as novel and efficient tools for atherosclerosis diagnosis. Biomed. Pharmacother. 93, 1098–1115. 10.1016/j.biopha.2017.07.012 28738519

[B60] Muhsin M. Graham J. Kirkpatrick P. (2004). Bevacizumab. Nat. Rev. Drug Discov. 3 (12), 995–996. 10.1038/nrd1601 15645606

[B61] Muñoz N. M. Williams M. Dixon K. Dupuis C. McWatters A. Avritscher R. (2021). Influence of injection technique, drug formulation and tumor microenvironment on intratumoral immunotherapy delivery and efficacy. J. Immunother. Cancer 9 (2), e001800. 10.1136/jitc-2020-001800 33589526 PMC7887346

[B62] Nakajima S. Koshino Y. Nomura T. Yamashita F. Agrawal S. Takakura Y. (2000). Intratumoral pharmacokinetics of oligonucleotides in a tissue-isolated tumor perfusion system. Antisense Nucleic Acid. Drug Dev. 10 (2), 105–110. 10.1089/oli.1.2000.10.105 10805161

[B63] Palombarini F. Di Fabio E. Boffi A. Macone A. Bonamore A. (2020). Ferritin nanocages for protein delivery to tumor cells. Molecules 25 (4), 825. 10.3390/molecules25040825 32070033 PMC7070480

[B64] Patel K. Patil A. Mehta M. Gota V. Vavia P. (2014). Oral delivery of paclitaxel nanocrystal (PNC) with a dual Pgp-CYP3A4 inhibitor: Preparation, characterization and antitumor activity. Int. J. Pharm. 472 (1), 214–223. 10.1016/j.ijpharm.2014.06.031 24954663

[B65] Patel S. K. Billingsley M. M. Frazee C. Han X. Swingle K. L. Qin J. (2022). Hydroxycholesterol substitution in ionizable lipid nanoparticles for mRNA delivery to T cells. J. Control. Release 347, 521–532. 10.1016/j.jconrel.2022.05.020 35569584 PMC9376797

[B66] Qiu X. Li S. Li X. Xiao Y. Li S. Fen Q. (2022). Experimental study of beta-TCP scaffold loaded with VAN/PLGA microspheres in the treatment of infectious bone defects. Colloids Surf. B Biointerfaces 213, 112424. 10.1016/j.colsurfb.2022.112424 35227993

[B67] Rezayan A. H. Mousavi M. Kheirjou S. Amoabediny G. Ardestani M. S. Mohammadnejad J. (2016). Monodisperse magnetite (Fe3O4) nanoparticles modified with water soluble polymers for the diagnosis of breast cancer by MRI method. J. Magnetism Magnetic Mater. 420, 210–217. 10.1016/j.jmmm.2016.07.003

[B68] Richardson N. H. Althouse S. K. Ashkar R. Cary C. Masterson T. Foster R. S. (2023). Late relapse of germ cell tumors after prior chemotherapy or surgery-only. Clin. Genitourin. Cancer S1558-7673, 00087-3. 10.1016/j.clgc.2023.03.018 37088659

[B69] Sarvepalli S. Parvathaneni V. Chauhan G. Shukla S. K. Gupta V. (2022). Inhaled indomethacin-loaded liposomes as potential therapeutics against non-small cell lung cancer (NSCLC). Pharm. Res. 39 (11), 2801–2815. 10.1007/s11095-022-03392-x 36109463

[B70] Schwechheimer C. Kuehn M. J. (2015). Outer-membrane vesicles from gram-negative bacteria: Biogenesis and functions. Nat. Rev. Microbiol. 13 (10), 605–619. 10.1038/nrmicro3525 26373371 PMC5308417

[B71] Shao Y. Xiang L. Zhang W. Chen Y. (2022). Responsive shape-shifting nanoarchitectonics and its application in tumor diagnosis and therapy. J. Control. Release 352, 600–618. 10.1016/j.jconrel.2022.10.046 36341936

[B72] Sharma A. Sharma N. Singh S. Dua K. (2023). Review on theranostic and neuroprotective applications of nanotechnology in multiple sclerosis. J. Drug Deliv. Sci. Technol. 81, 104220. 10.1016/j.jddst.2023.104220

[B73] Shelar S. B. Barick K. C. Dutta B. Basu M. Hassan P. A. (2023). Selective targeting of gold nanoparticles for radiosensitization of somatostatin 2 receptor-expressing cancer cells. J. Drug Deliv. Sci. Technol. 82, 104381. 10.1016/j.jddst.2023.104381

[B74] Shi W. Wan X. Wang Y. He J. Huang X. Xu Y. (2023). Nanoparticle albumin-bound paclitaxel-based neoadjuvant regimen: A promising treatment option for HER2-low-positive breast cancer. Nanomedicine 49, 102666. 10.1016/j.nano.2023.102666 36889422

[B75] Singh S. Meena A. Luqman S. (2021). Baicalin mediated regulation of key signaling pathways in cancer. Pharmacol. Res. 164, 105387. 10.1016/j.phrs.2020.105387 33352232

[B76] Snow A. Lin L. Hunsucker S. A. Wang Y. Liu R. Armistead P. M. (2022). Development of a mRNA lipid nanoparticle (mRNA-LNP) cancer vaccine to prevent leukemia relapse after stem cell transplant. Blood 140, 7382–7383. 10.1182/blood-2022-160218

[B77] Spada A. Emami J. Tuszynski J. A. Lavasanifar A. (2021). The uniqueness of albumin as a carrier in nanodrug delivery. Mol. Pharm. 18 (5), 1862–1894. 10.1021/acs.molpharmaceut.1c00046 33787270

[B78] Spigel D. R. Jotte R. M. Aix S. P. Gressot L. Morgensztern D. McCleod M. (2021). Nanoparticle albumin-bound paclitaxel plus carboplatin induction followed by nanoparticle albumin-bound paclitaxel maintenance in squamous non–small-cell lung cancer (ABOUND.sqm): A phase III randomized clinical trial. Clin. Lung Cancer 22 (1), 6–15.e4. 10.1016/j.cllc.2020.09.007 33097414

[B79] Sun X. Wang G. Zhang H. Hu S. Liu X. Tang J. (2018). The blood clearance kinetics and pathway of polymeric micelles in cancer drug delivery. ACS Nano 12 (6), 6179–6192. 10.1021/acsnano.8b02830 29847730

[B80] Tahtinen S. Tong A. J. Himmels P. Oh J. Paler-Martinez A. Kim L. (2022). IL-1 and IL-1ra are key regulators of the inflammatory response to RNA vaccines. Nat. Immunol. 23 (4), 532–542. 10.1038/s41590-022-01160-y 35332327

[B81] Tan B. Wu Y. Wu Y. Shi K. Han R. Li Y. (2021). Curcumin-Microsphere/IR820 hybrid bifunctional hydrogels for *in situ* osteosarcoma chemo-co-thermal therapy and bone reconstruction. ACS Appl. Mater Interfaces 13 (27), 31542–31553. 10.1021/acsami.1c08775 34191477

[B82] Tavallaii A. Meybodi K. T. Nejat F. Habibi Z. (2023). Current status of research on targeted therapy against central nervous system tumors in low- and lower-middle-income countries. World Neurosurg. 174, 74–80. 10.1016/j.wneu.2023.03.030 36918096

[B83] Teja P. K. Mithiya J. Kate A. S. Bairwa K. Chauthe S. K. (2022). Herbal nanomedicines: Recent advancements, challenges, opportunities and regulatory overview. Phytomedicine 96, 153890. 10.1016/j.phymed.2021.153890 35026510

[B84] Thangavel K. Lakshmikuttyamma A. Thangavel C. Shoyele S. A. (2022). CD44-targeted, indocyanine green-paclitaxel-loaded human serum albumin nanoparticles for potential image-guided drug delivery. Colloids Surf. B Biointerfaces 209 (1), 112162. 10.1016/j.colsurfb.2021.112162 34752986

[B85] Truffi M. Fiandra L. Sorrentino L. Monieri M. Corsi F. Mazzucchelli S. (2016). Ferritin nanocages: A biological platform for drug delivery, imaging and theranostics in cancer. Pharmacol. Res. 107, 57–65. 10.1016/j.phrs.2016.03.002 26968122

[B86] Wang B. Zhang W. Zhou X. Liu M. Hou X. Cheng Z. (2019a). Development of dual-targeted nano-dandelion based on an oligomeric hyaluronic acid polymer targeting tumor-associated macrophages for combination therapy of non-small cell lung cancer. Drug Deliv. 26 (1), 1265–1279. 10.1080/10717544.2019.1693707 31777307 PMC6896416

[B87] Wang D. Lippard S. J. (2005). Cellular processing of platinum anticancer drugs. Nat. Rev. Drug Discov. 4 (4), 307–320. 10.1038/nrd1691 15789122

[B88] Wang L. Wang C. Tao Z. Zhao L. Zhu Z. Wu W. (2019b). Curcumin derivative WZ35 inhibits tumor cell growth via ROS-YAP-JNK signaling pathway in breast cancer. J. Exp. Clin. Cancer Res. 38 (1), 460. 10.1186/s13046-019-1424-4 31703744 PMC6842168

[B89] Wang R. Sun Y. He W. Chen Y. Lu E. Sha X. (2021). Pulmonary surfactants affinity Pluronic-hybridized liposomes enhance the treatment of drug-resistant lung cancer. Int. J. Pharm. 607, 120973. 10.1016/j.ijpharm.2021.120973 34391853

[B90] Wang Y. Douglas T. (2021). Protein nanocage architectures for the delivery of therapeutic proteins. Curr. Opin. Colloid & Interface Sci. 51, 101395. 10.1016/j.cocis.2020.101395

[B91] Wang Y. Hu J. K. Krol A. Li Y. P. Li C. Y. Yuan F. (2003). Systemic dissemination of viral vectors during intratumoral injection. Mol. Cancer Ther. 2 (11), 1233–1242. 14617797

[B92] Wang Z. Ma W. Fu X. Qi Y. Zhao Y. Zhang S. (2023). Development and applications of mRNA treatment based on lipid nanoparticles. Biotechnol. Adv. 65, 108130. 10.1016/j.biotechadv.2023.108130 36933868

[B93] Wang Z. Zhao Y. Yang Z. Li X. Xing H. Qu W. (2022). Construction of intelligent responsive drug delivery system and multi-mode imaging based on gold nanodots. Macromol. Rapid Commun. 43 (10), e2200034. 10.1002/marc.202200034 35332623

[B94] Wei L. Ji Y. Gong W. Kang Z. Meng M. Zheng A. (2015). Preparation, physical characterization and pharmacokinetic study of paclitaxel nanocrystals. Drug Dev. Ind. Pharm. 41 (8), 1343–1352. 10.3109/03639045.2014.950272 25156484

[B95] Wu H. Lv W. H. Zhu Y. Y. Jia Y. Y. Nie F. (2023). Ultrasound-mediated mesoporous silica nanoparticles loaded with PDLIM5 siRNA inhibit gefitinib resistance in NSCLC cells by attenuating EMT. Eur. J. Pharm. Sci. 182, 106372. 10.1016/j.ejps.2023.106372 36621614

[B96] Xiao Y. Li X. Mao J. Zheng H. Ji R. Wang Z. (2022). Reverse anti-breast cancer drug resistance effects by a novel two-step assembled nano-celastrol medicine. Nanoscale 14 (21), 7856–7863. 10.1039/d2nr02064e 35583119

[B97] Xiao Y. Liu J. Guo M. Zhou H. Jin J. Liu J. (2018). Synergistic combination chemotherapy using carrier-free celastrol and doxorubicin nanocrystals for overcoming drug resistance. Nanoscale 10 (26), 12639–12649. 10.1039/c8nr02700e 29943786

[B98] Xu B. Zeng F. Deng J. Yao L. Liu S. Hou H. (2023). A homologous and molecular dual-targeted biomimetic nanocarrier for EGFR-related non-small cell lung cancer therapy. Bioact. Mater 27, 337–347. 10.1016/j.bioactmat.2023.04.005 37122898 PMC10140750

[B99] Yang Y. Huang Z. Li J. Mo Z. Huang Y. Ma C. (2019). PLGA porous microspheres dry powders for codelivery of afatinib-loaded solid lipid nanoparticles and paclitaxel: Novel therapy for EGFR tyrosine kinase inhibitors resistant nonsmall cell lung cancer. Adv. Healthc. Mater 8 (23), e1900965. 10.1002/adhm.201900965 31664795

[B100] Yardley D. A. (2013). nab-Paclitaxel mechanisms of action and delivery. J. Control. Release 170 (3), 365–372. 10.1016/j.jconrel.2013.05.041 23770008

[B101] Yimit A. Adebali O. Sancar A. Jiang Y. (2019). Differential damage and repair of DNA-adducts induced by anti-cancer drug cisplatin across mouse organs. Nat. Commun. 10 (1), 309. 10.1038/s41467-019-08290-2 30659176 PMC6338751

[B102] Yoneshima Y. Morita S. Ando M. Nakamura A. Iwasawa S. Yoshioka H. (2021). Phase 3 trial comparing nanoparticle albumin-bound paclitaxel with docetaxel for previously treated advanced NSCLC. J. Thorac. Oncol. 16 (9), 1523–1532. 10.1016/j.jtho.2021.03.027 33915251

[B103] Yoo J. W. Irvine D. J. Discher D. E. Mitragotri S. (2011). Bio-inspired, bioengineered and biomimetic drug delivery carriers. Nat. Rev. Drug Discov. 10 (7), 521–535. 10.1038/nrd3499 21720407

[B104] Yu M. Zhang C. Tang Z. Tang X. Xu H. (2019). Intratumoral injection of gels containing losartan microspheres and (PLG-g-mPEG)-cisplatin nanoparticles improves drug penetration, retention and anti-tumor activity. Cancer Lett. 442, 396–408. 10.1016/j.canlet.2018.11.011 30439541

[B105] Zeng Y. Escalona-Rayo O. Knol R. Kros A. Slutter B. (2023). Lipid nanoparticle-based mRNA candidates elicit potent T cell responses. Biomaterials Sci. 11 (3), 964–974. 10.1039/d2bm01581a 36537916

[B106] Zhang J. Xu L. Hu H. Chen E. (2022a). The combination of MnO(2)@Lipo-coated gefitinib and bevacizumab inhibits the development of non-small cell lung cancer. Drug Deliv. 29 (1), 466–477. 10.1080/10717544.2022.2032872 35147070 PMC8843201

[B107] Zhang L. Liu Z. Kong C. Liu C. Yang K. Chen H. (2018). Improving drug delivery of micellar paclitaxel against non-small cell lung cancer by coloading Itraconazole as a micelle stabilizer and a tumor vascular manipulator. Small 14 (51), e1802112. 10.1002/smll.201802112 30444572

[B108] Zhang L. Loh X. J. Ruan J. (2022b). Photoelectrochemical nanosensors: An emerging technique for tumor liquid biopsy. J. Photochem. Photobiol. A Chem. 429, 113942. 10.1016/j.jphotochem.2022.113942

[B109] Zhang L. Wang H. Zhao G. Li N. Wang X. Li Y. (2021). Anti-Tim4 grafting strongly hydrophilic metal-organic frameworks immunoaffinity flake for high-efficiency capture and separation of exosomes. Anal. Chem. 93 (16), 6534–6543. 10.1021/acs.analchem.1c00528 33851819

[B110] Zhang P. Xiao Y. Sun X. Lin X. Koo S. Yaremenko A. V. (2023). Cancer nanomedicine toward clinical translation: Obstacles, opportunities, and future prospects. Med 4 (3), 147–167. 10.1016/j.medj.2022.12.001 36549297

[B111] Zong Y. Lin Y. Wei T. Cheng Q. (2023). Lipid nanoparticle (LNP) enables mRNA delivery for cancer therapy. Adv. Mater. Deerf. Beach, Fla) 2023, e2303261. 10.1002/adma.202303261 37196221

